# Restoring autophagic flux attenuates cochlear spiral ganglion neuron degeneration by promoting TFEB nuclear translocation via inhibiting MTOR

**DOI:** 10.1080/15548627.2019.1569926

**Published:** 2019-02-01

**Authors:** Bin Ye, Quan Wang, Haixia Hu, Yilin Shen, Cui Fan, Penghui Chen, Yan Ma, Hao Wu, Mingliang Xiang

**Affiliations:** a Department of Otolaryngology & Head and Neck Surgery, Ruijin Hospital, Shanghai Jiao Tong University School of Medicine, Shanghai, China; b Shanghai Key Laboratory of Translational Medicine on Ear and Nose diseases, The Ninth People's Hospital, Shanghai Jiao Tong University School of Medicine, Shanghai, China; c Ear Institute, Shanghai Jiao tong University School of Medicine, Shanghai, China

**Keywords:** Autophagy dysfunction, degeneration, MTOR pathway, oxidative stress, spiral ganglion neuron, TFEB

## Abstract

Macroautophagy/autophagy dysfunction is associated with many neurodegenerative diseases. TFEB (transcription factor EB), an important molecule that regulates lysosomal and autophagy function, is regarded as a potential target for treating some neurodegenerative diseases. However, the relationship between autophagy dysfunction and spiral ganglion neuron (SGN) degeneration and the role of TFEB in SGN degeneration has not yet been established. Here, we showed that in degenerated SGNs, induced by sensory epithelial cell loss in the cochlea of mice following kanamycin and furosemide administration, the lipofuscin area and oxidative stress level were increased, the nuclear-to-cytoplasmic TFEB ratio was decreased, and the late stage of autophagic flux was impaired. After autophagy dysfunction was partially ameliorated with an MTOR inhibitor, which promoted TFEB translocation into the nucleus from the cytoplasm, we found that the lysosomal deficits were significantly relieved, the oxidative stress level was reduced, and the density of surviving SGNs and auditory nerve fibers was increased. The results in the present study reveal that autophagy dysfunction is an important component of SGN degeneration, and TFEB may be a potential target for attenuating SGN degeneration following sensory epithelial cell loss in the cochlea of mice.

**Abbreviations**: 3-NT: 3-nitrotyrosine; 4-HNE: 4-hydroxynonenal; 8-OHdG: 8-hydroxy-2ʹ-deoxyguanosine; ABR: auditory brainstem response; APP: amyloid beta (A4) precursor protein; CLEAR: coordinated lysosomal expression and regulation; CTSB: cathespin B; CTSD: cathespin D; SAMR1: senescence-accelerated mouse/resistance 1; SAMP8: senescence-accelerated mouse/prone 8; MAPK1/ERK2: mitogen-activated protein kinase 1; MTOR: mechanistic target of rapamycin kinase; SGN: spiral ganglion neuron; SQSTM1/p62: sequestosome 1; TEM: transmission electron microscope; TFEB: transcription factor EB

## Introduction

Sensorineural hearing loss is a common sensory disease that seriously affects physical and mental health and quality of life in humans. Although the pathogenic factors are diverse, the underlying pathological features of sensorineural hearing loss are similar: sensory epithelial cell (hair cell and support cell) loss and the progressive degeneration of cochlear nerve fibers and spiral ganglion neurons (SGNs). However, many pathogenic factors resulting in sensorineural hearing loss mainly cause sensory epithelial cells to be destroyed in the cochlea and do not cause simultaneous SGN injuries. As a consequence of cochlear sensory epithelial cell loss, the degeneration of cochlear nerve fibers and SGNs is, in essence, atrophy from disuse, which results from the loss of the target organ and neurotrophic factors [1]. With the aid of stem cell transplantation [2], gene rescue [3], neurotrophic factor inner ear administration [1] and other means, hair cell regeneration has already been achieved in many scientific experiments. Hair cell regeneration in vivo is meaningless if the regenerated hair cells cannot be innervated by SGNs in the cochlea. Cochlear implants are currently an effective tool for restoring hearing function in patients with severe sensorineural hearing loss. The cochlear implants replace hair cells, converting external sound signals into electrical impulses to directly stimulate cochlear nerve fibers and SGNs, thereby reconstructing the hearing pathway. The number of surviving SGNs determines the effect of cochlear implantation. Therefore, to achieve better hearing rehabilitation via cochlear implantation, a required number of surviving SGNs is necessary [2,3]. To rehabilitate hearing diminished by sensorineural hearing loss, preventing or alleviating the degeneration of cochlear nerve fibers and SGNs following sensory epithelial cell loss is crucial.

Due to a lack of acoustic signal stimulation and neurotrophic factors, SGNs gradually undergo morphological changes after sensory epithelial cell damage in the organ of Corti [1], including retreating axons, degeneration from auditory nerve terminals to neuronal soma, and finally neuronal death. This retrograde degeneration of SGNs resulting from sensory epithelial cell loss is similar to the dying-back process, a pathological pattern that occurs in many neurodegenerative diseases. Neurotrophic factors are crucial for the survival of SGNs and their nerve fibers. Konstantina et al. found that decreasing NT-3 expression in support cells of adult mice by suppressing the ERBB4 signal results in the death of 80% of type I SGNs, although no significant changes in morphology are observed in the supporting cells or hair cells [4]. Neurotrophin administration [5,6], electrical stimulation [7,8] and embryonic stem cell transplantation [9] have been employed by some researchers to reduce SGN loss resulting from inner ear damage. Miller et al. used kanamycin and ethacrynic acid to destroy sensory epithelial cells in the inner ear of guinea pigs to establish a SGN degeneration model. Continuous infusion of BDNF into the scala tympani for 2 weeks preserves SGN survival [5]. Furthermore, Staecker et al. reported that the number of SGNs is significantly increased 4 weeks later after an exogenous *Bdnf* gene is transfected into the cochlea of CBA/6J mice in which hair cell loss and SGN degeneration is induced with cochlear injection of neomycin [1]. After neonatally deafened cats induced by neomycin received unilateral electrical stimulation for 5–12 months, SGN density ipsilateral to the ear that received the electrical stimulation is within approximately half a percent of the density in a normal ear, which is 20% higher than the density in the side contralateral to the stimulation [8]. Corrales et al. used ouabain to injure the SGNs of adult gerbils and then transplanted embryonic stem cells into the cochlear modiolus of the animal. The researchers found that, with time, the stem cells were able to project new neurites to the denervated organ of Corti [9]. Although the results of the above experiments were encouraging, the administrations employed to save SGNs not only were unfavorable to the cochlear microenvironment but also may destroy the residual hearing capacity. Therefore, more ideal means to prevent SGNs from degeneration are needed.

Autophagy can remove intracellular aggregate proteins and damaged organelles to maintain homeostasis, thus preventing cell degeneration and death. Because neuron regeneration seems impossible in vivo, this ‘self-cleaning’ mechanism is particularly critical for neurons to maintain their physiological function and survive. Recent studies have shown that impaired autophagy plays a crucial role in the development and progression of neurodegenerative diseases. For many neurodegenerative diseases, such as Alzheimer disease (AD), Parkinson disease (PD), and Huntington disease (HD), a common pathological basis is impaired autophagy-lysosomal pathways induced by toxic protein accumulation in neurons [10–12]. For example, BECN1, a crucial protein at the initial phase of autophagy, is significantly reduced in the impaired brain area of early-stage AD patients and mice, and the accumulation of APP (amyloid beta precursor protein) in the neurons of APP transgenic mice results in neurodegeneration [11]. In addition, in HD cell and mouse models, the accumulation of the pathogenic protein HTT (huntingtin) causes neurodegeneration and eventually HD by binding with BECN1 to decrease autophagy levels [13]. In normal neurons, PINK1 is bound to BECN1 to increase autophagy levels, which upregulates aberrant protein clearance and reduces neurodegeneration [12]. Mutations in the autophagy-related gene *PINK1* have been implicated as crucial factors in Parkinson disease, and decreased autophagy levels promote the development of neurodegenerative diseases [11,14].

Some studies have demonstrated that autophagy might participate in SGN development [15] and that an increased autophagy level might occur in injured SGNs in an in vitro model [16]. Previous studies that examined the relationship between autophagy and inner ear disorders were mainly focused on the role of autophagy in hair cell damage [17,18]. To our knowledge, the role of autophagy in the progressive degeneration of SGNs, especially the effect of ameliorating autophagy dysfunction in SGN degeneration, has not been reported. In this study, for the first time, we found that autophagic flux was impaired and that lysosomal capacity was decreased during the initial stages of SGN degeneration in the mouse cochlea. The transcription factor TFEB, which regulates lysosomal and autophagic function, was significantly arrested in the cytoplasm. With the aid of an MTOR inhibitor, temsirolimus (CCI-779), we promoted TFEB translocation from the cytoplasm into the nucleus; thus, we partially restored autophagy and lysosomal function and reduced oxidative stress, ultimately attenuating SGN and nerve fiber degeneration.

## Results

### A mouse model of SGN degeneration was successfully established via the destruction of cochlear sensory epithelial cells

**Figure 1.**
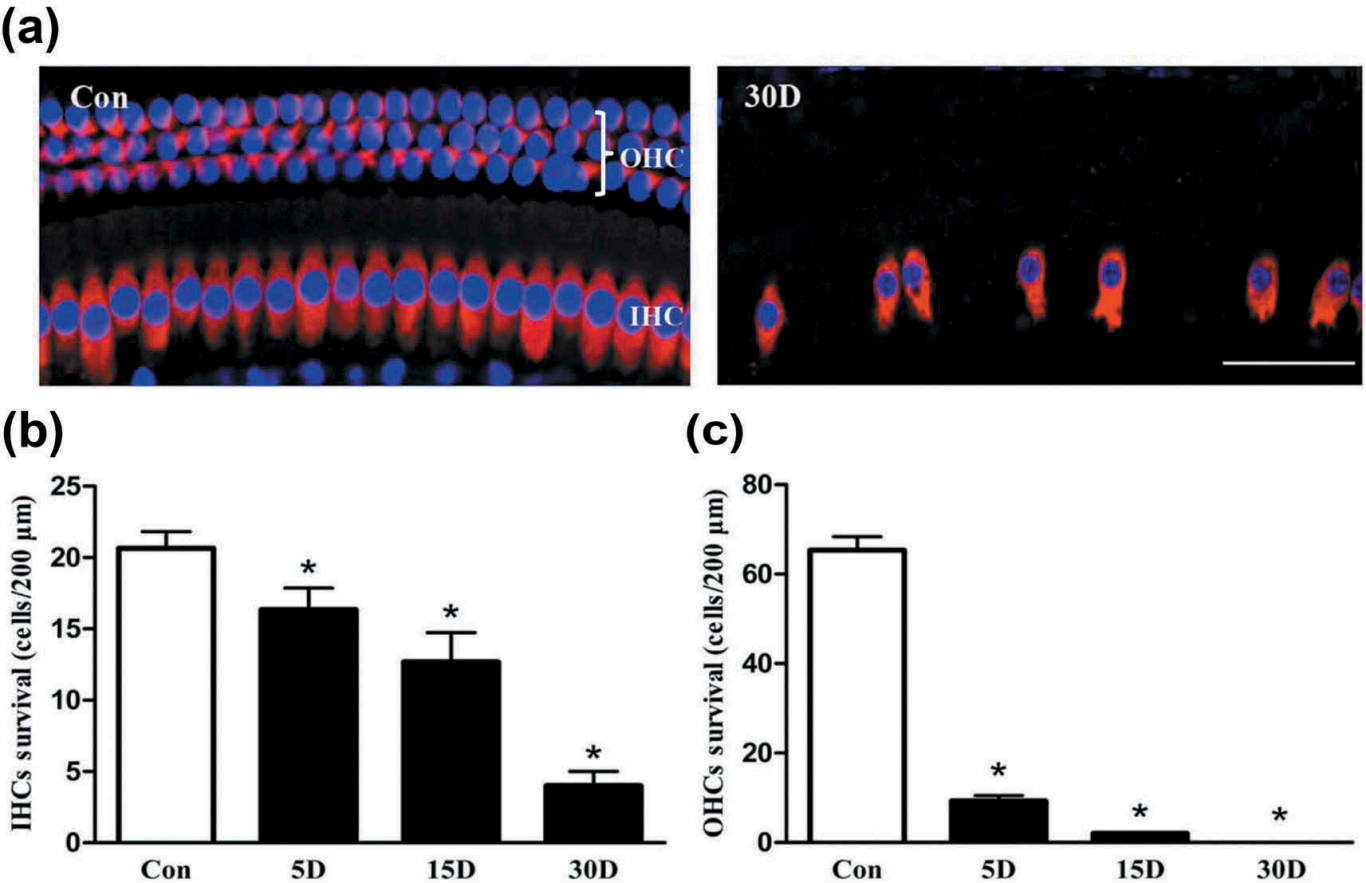
Sensory epithelial cells were damaged in the cochlea of mice after kanamycin and furosemide administration. (a) Morphological changes of the hair cells in the cochlea of mice from the normal and experimental groups, as determined under a confocal microscope. Images were taken from the middle turn of cochlea. Con, normal mice without drug treatment. Three rows of outer hair cells and 1 row of inner hair cells in the cochlea of mice were neatly arranged, and no hair cells were lost. 30D, 30 days after drug administration. The arrangement of hair cells was unorganized, and hair cell loss was evident. (b and c) The density of the residual inner or outer hair cells in the middle turn of cochlea at each time point. *, Compared with that in the blank control group, the density of hair cells was significantly lower (*P* < 0.05). Data are represented as the means ± SD; n = 5. OHC, outer hair cell; IHC, Inner hair cell; red, MYO7A staining; blue, DAPI staining. Scale bar: 40 µm.

**Figure 2.**
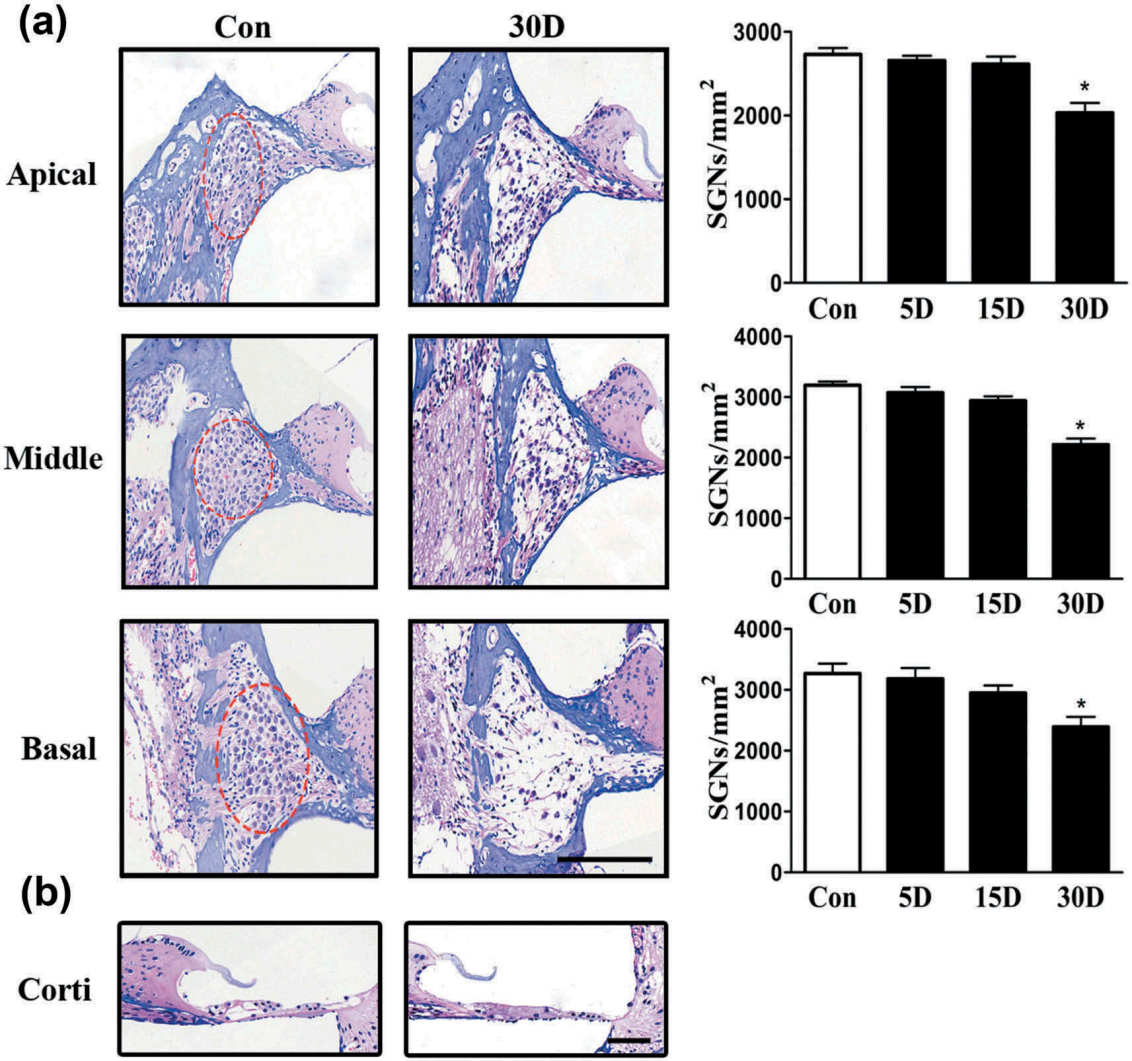
SGN degeneration in the cochlea of mice was successfully established following the destruction of cochlear sensory epithelial cells. (a) Morphological changes of SGNs (red circle) in the apical, middle, and basal turn of the cochlea from the normal and experimental group, as determined with HE staining under a light microscope. The quantified SGN density in each turn is shown on the right panel. In the experimental group, the SGN density progressively decreased. (b) Morphological changes of the organ of Corti taken from the middle turn of cochlea from the normal and experimental group. The sensory epithelial cells and tunnel of Corti were disrupted and collapsed in the experimental groups. *, compared with that in the blank control group, the SGN density was significantly lower (*P* < 0.05). Data are represented as the means ± SD; n = 6 for each time point. Con, normal mice without drug treatment; 30D, 30 days after the end of the drug administration. Scale bar: 100 µm.

Morphological observations (Figures 1 and 2) showed that a single injection of kanamycin sulfate (1 g/kg) and an intraperitoneal injection of furosemide (0.4 g/kg), which was injected 30 ~ 45 min after the kanamycin injection, successfully induced SGN degeneration in the cochlea of mice via the destruction of cochlear sensory epithelial cells, which was consistent with the results of our previous studies [19,20]. The structure of the organ of Corti was completely collapsed and disintegrated, nearly no sensory epithelial cells remained on the 30th day after kanamycin and furosemide administration, and the basilar membrane was covered by a continuous layer of flattened cubic epithelial cells.

### Impaired autophagy occurred during SGN degeneration in mice

**Figure 3.**
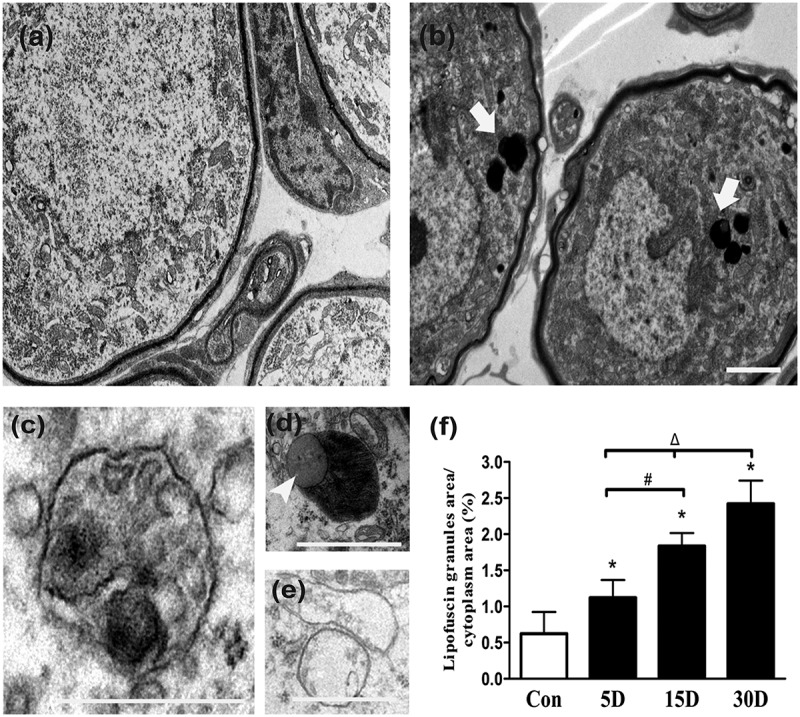
Ultrastructural observation of lipofuscins in the cochlear spiral ganglion neurons of mice. (a) Ultrastructure features of the SGNs in normal mice (8 w). (b) Thirty days after administration, lipofuscins (white arrows) of degenerated SGNs aggregated progressively. (c) Autophagic vacuole. (d) Intracellular inclusion body (white arrowhead) containing lipid droplets. (e) Enlarged and cristae-disrupted mitochondria. (f) The lipofuscin area in the degenerated SGN cytoplasm gradually increased with the progression of neurodegeneration. *, compared with that in the blank control group, the lipofuscin area within the degenerated SGN cytoplasm was significantly increased (*P* < 0.05); #, compared with that on the 5th day after ototoxic drug administration, the lipofuscin area within the degenerated SGN cytoplasm on the 15th day was significantly higher (*P < *0.05); Δ, compared with the area at the previous observation point, the area of lipofuscin within the degenerated SGN cytoplasm was further increased by the 30th day after ototoxic drug administration (*P* < 0.05). Con, normal mice without drug treatment; 30D, 30 days after drug administration. Images of TEM were taken from the middle turn of cochlea. Scale bar: 1 µm.

**Figure 4.**
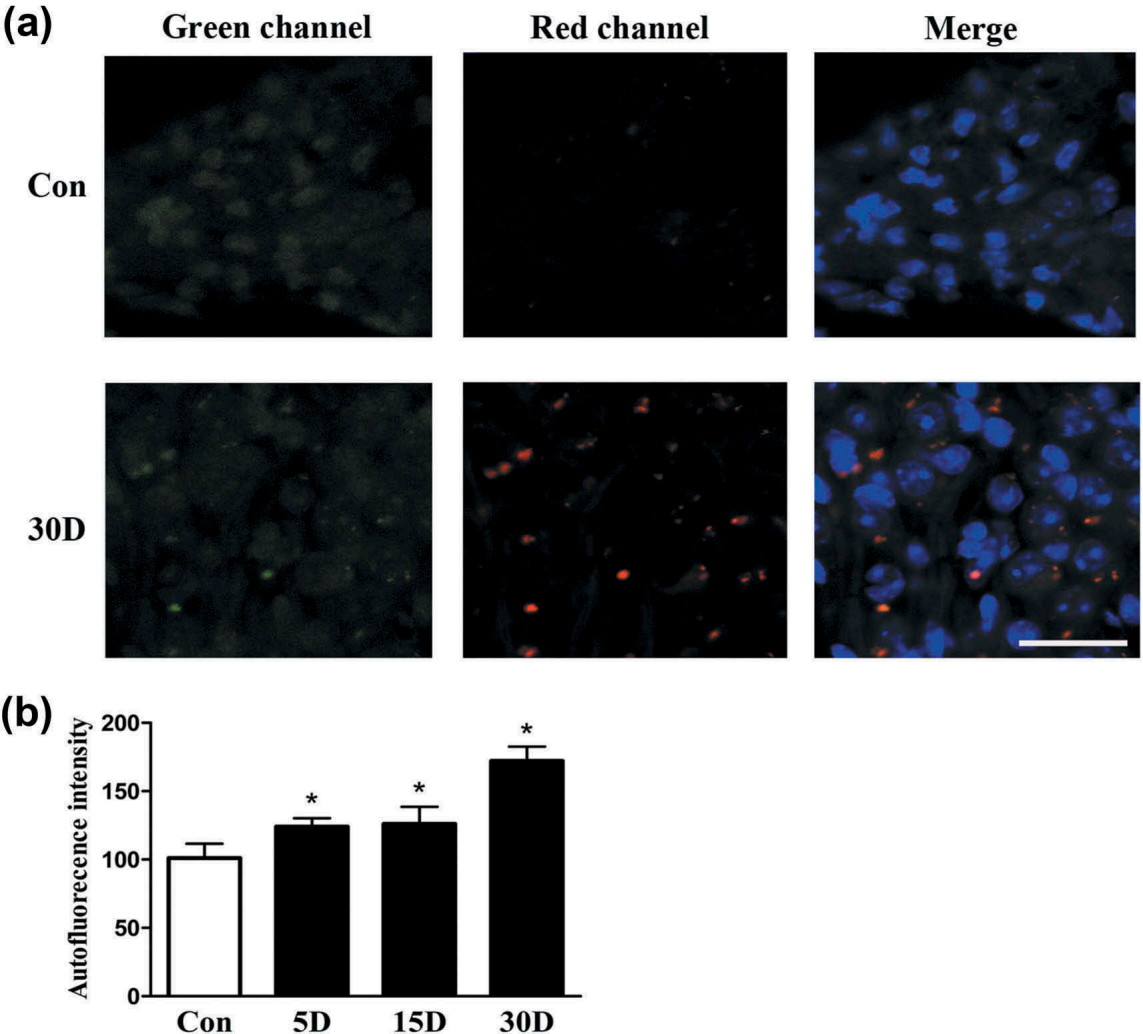
Lipofuscin autofluorescence was increased during SGN degeneration in the cochleae of mice. (a) Lipofuscin autofluorescence within SGNs taken from the middle turn of cochlea in the blank control (Con) and experimental mice on the 30th day after ototoxic drug administration, as observed under a confocal microscope. Lipofuscin-like granules are represented as yellow dots in the merged panel. (b) Lipofuscin autofluorescence intensity within SGNs at each observation point. *, compared with that in the blank control group, the autofluorescence intensity in the degenerated SGNs was statistically increased (*P* < 0.05).; con normal mice without drug treatment; 30D, 30 days after drug administration. Scale bar: 50 µm.

By transmission electron microscopy (TEM) analysis, we found lipofuscin aggregation and autophagic vacuole formation in the soma of degenerated SGNs. Because SGN degeneration was aggravated following sensory epithelial cell loss in the cochleae of mice, lipofuscin gradually accumulated in the SGNs (Figure 3(a,b)), and the area of the cytoplasm occupied by lipofuscin granules increased (Figure 3(f)). Autophagic vacuoles and lipid droplet-like intracellular inclusion bodies were formed (Figure 3(c,d)). Mitochondria were swollen, and mitochondrial cristae disappeared or became vacuolated (Figure 3(e)). Autofluorescence is a characteristic of lipofuscin [21,22]; therefore, a confocal microscope was used to observe the appearance of autofluorescence within SGNs. We found that spontaneous fluorescence in degenerated SGNs was significantly stronger than that in normal SGNs and was gradually enhanced as SGN degeneration progressed (Figure 4(a,b)). These results confirmed our TEM observation that lipofuscin was increased in degenerated SGNs. Lipofuscin is composed of autolysosome remnants that are formed by lipid peroxidation products, accumulated proteins, and other debris that are not completely hydrolyzed by autolysosomes. Therefore, the progressive aggregation of lipofuscin probably demonstrated that autophagy was impaired in degenerated SGNs [22,23].

### Autophagic flux was impaired during SGN degeneration

**Figure 5.**
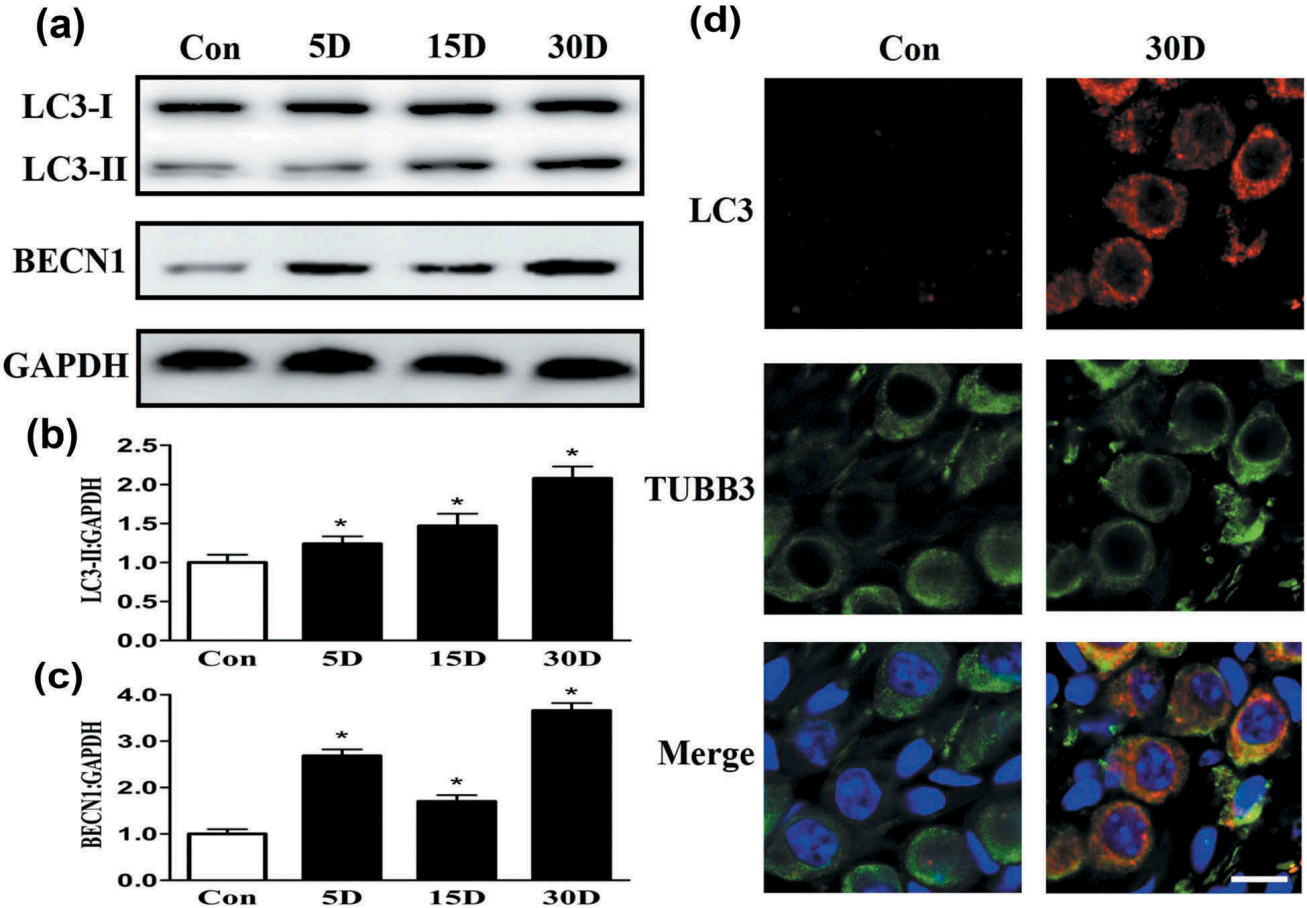
The autophagy level was increased in degenerated mouse SGNs. (a-c) Western blot results revealed that the levels of the autophagy-related proteins LC3 and BECN1 were increased in the degenerated SGNs on the 5th, 15th and 30th day after ototoxic drug administration and were significantly different from those in the normal mice. *, *P* < 0.05. (d) Immunofluorescence staining of LC3 puncta (red) also demonstrated that the LC3 level in the degenerated SGNs (green) was significantly increased on the 30th day after drug administration. Con, normal mice without drug treatment; 30D, 30 days after drug administration. Images of immunofluorescence staining were taken from the middle turn of cochlea. Scale bar: 10 µm.

**Figure 6.**
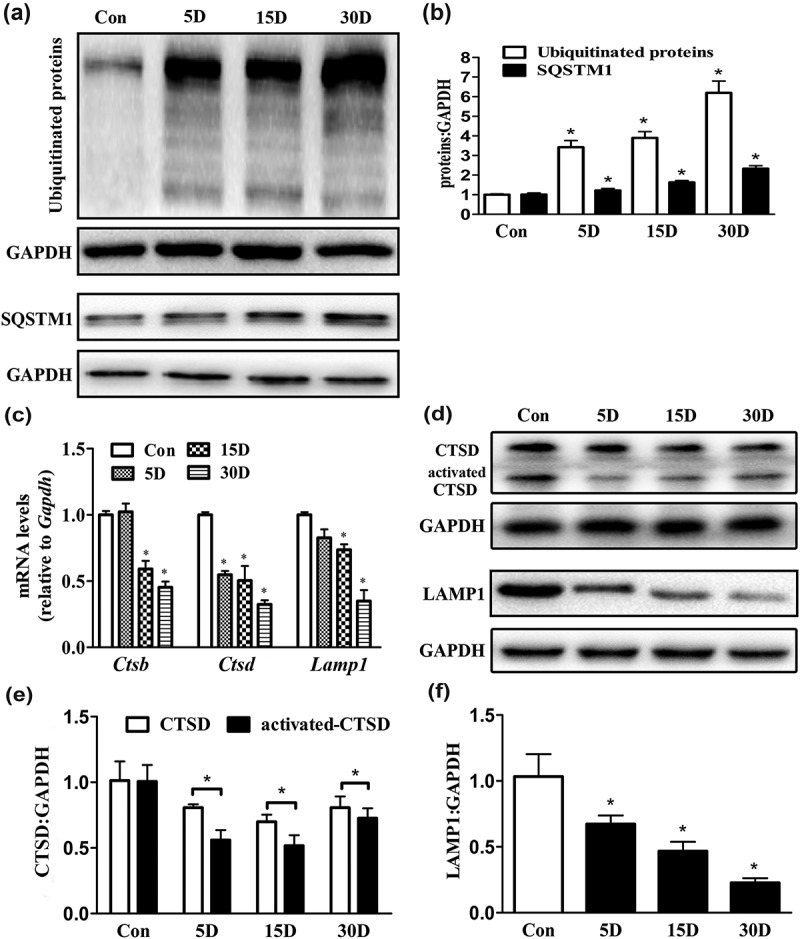
Autophagic flux and lysosomal hydrolase levels were impaired during the process of SGN degeneration in the cochlea of mice. (a and b) Western blot analysis revealed that levels of the autophagic cargo receptor protein SQSTM1 and ubiquitinated proteins were increased in SGNs on the 5th, 15th and 30th day after ototoxic drug administration. (c) Quantitative real-time PCR results revealed that compared with that in the blank control group, the *Ctsd* level was significantly decreased on the 5th day after the ototoxic drugs were given. By the 15th day, the levels of *Lamp1, Ctsb* and *Ctsd* were significantly lower in the treated mice than in the normal mice (*P* < 0.05). On the 30th day after ototoxic drug administration, the levels of *Ctsd* and *Lamp1* were further decreased (*P* < 0.05). *Gapdh* was used as the internal reference. (d-f) Western blot assays revealed that the levels of LAMP1 and CTSD were significantly decreased with SGN degeneration compared with these levels in the blank control SGNs. *, *P* < 0.05; Con, normal mice without drug treatment; 5D, 15D, and 30D, 5, 15, and 30 days after drug administration, respectively.

Because autophagy impairment probably occurred during SGN degeneration, proteins associated with autophagic flux, such as LC3, BECN1, SQSTM1, and ubiquitinated proteins were assessed. The results revealed that the levels of LC3-II and BECN1, proteins associated with autophagy initiation (Figure 5(a-c)), were significantly higher on the 5th, 15th and 30th day after drug administration than the corresponding levels in the blank control group (*P* < 0.05). Immunofluorescence staining also showed that the immunofluorescence accumulation of LC3 gradually increased as SGN degeneration progressed (Figure 5(d)). Next, we detected the cargo receptor protein SQSTM1, which is indicative of the level of autophagic degradation. Western blotting analysis revealed that SQSTM1 accumulation was significantly higher in degenerated SGNs than in blank control SGNs (*P* < 0.05) (Figure 6(a,b)). To confirm that autophagic degradation was impaired, we also examined ubiquitinated proteins, which are also critical markers of autophagic degradation. The results (Figure 6(a,b)) were similar to those of SQSTM1. These results demonstrated that during SGN degeneration, the autophagic flux of degenerated neurons was indeed damaged.

### Lysosome and hydrolase levels were significantly decreased during SGN degeneration

Recently, studies have reported that lysosomal dysfunction is the primary cause of autophagy deficits in many neurodegenerative diseases [14,24,25]. In our study, autophagy flux arrest and lipofuscin accumulation were distinct during SGN degeneration, and autophagy initiation was not decreased. Therefore, the terminal degradation executors of autophagic vacuoles, lysosomes and their internal hydrolases were investigated. Using real-time polymerase chain reaction (PCR), we detected the levels of the lysosomal membrane and hydrolase proteins encoded by the genes *Lamp1, Ctsb* and *Ctsd*. The results revealed that on the 5th day after ototoxic drug administration, the *Lamp1* and *Ctsb* levels in the degenerated SGNs were not significantly different from those in the normal SGNs (Figure 6(c)). On the 15th day, the *Ctsb, Ctsd* and *Lamp1* levels in the degenerated SGNs were significantly less than those in the blank control SGNs (*P* < 0.05), and by the 30th day, the levels of *Ctsb, Ctsd* and *Lamp1* were decreased even further. Then, we detected the levels of LAMP1 and CTSD by western blotting. The results of the western blot assay showed that LAMP1 and activated CTSD levels were significantly lower in the degenerated SGNs than in the blank control SGNs (Figure 6(d-f), *P* < 0.05). These results demonstrated that lysosome and hydrolase levels were significantly decreased in the degenerated SGNs.

### TFEB was arrested in the cytoplasm during SGN degeneration

**Figure 7.**
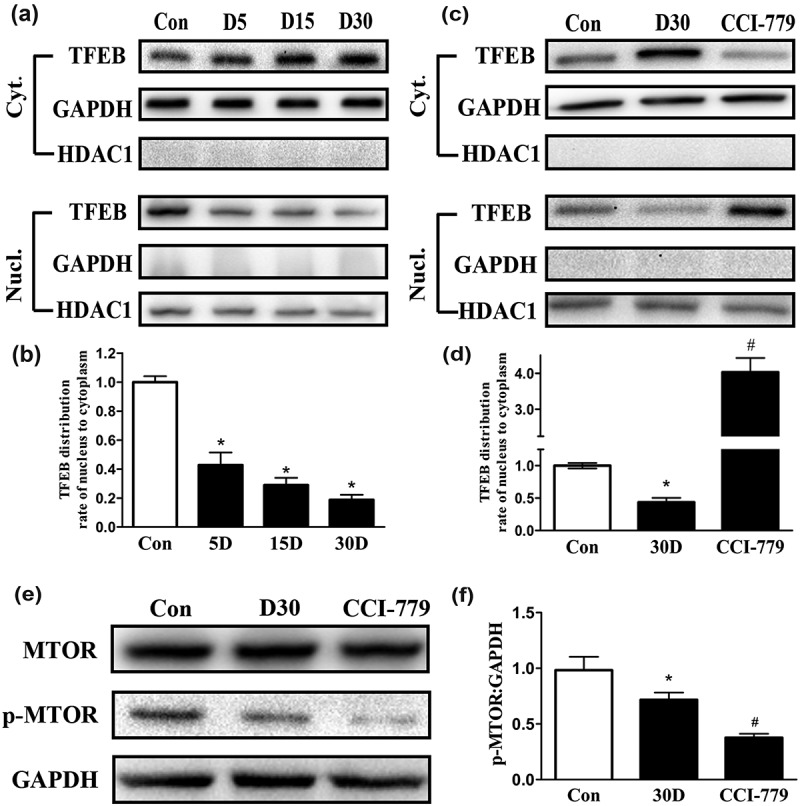
TFEB nuclear translocation was promoted in the degenerated SGNs via MTOR pathway inhibition by CCI-779 intervention. (a and b) Western blot analysis revealed that the presence of TFEB in the nucleus of degenerated SGNs gradually decreased on the 5th, 15th and 30th day after ototoxic drug administration, while the TFEB level increased in the cytoplasm. (c and d) Compared with that of the blank control and negative control groups, the TFEB nucleus-to-cytoplasm distribution ratio was significantly higher in the degenerated SGNs of the experimental group based on western blot. (e and f) The p-MTOR level was significantly suppressed in the experimental group after the mice were treated with CCI-779. *, the difference between the blank control group and the negative control group was significant (*P* < 0.05); #, compared with the blank control or negative control group, the ratio was significantly increased in the experimental group (*P* < 0.05); CCI-779, experimental group; 30D, negative control group; con, blank control group.

Due to the significant decrease in the lysosome and hydrolase levels of the degenerated SGNs, TFEB (transcription factor EB), a major regulator of the coordinated lysosomal expression and regulation (CLEAR) network, was investigated. Recently, many studies have reported that TFEB is a key transcription factor involved in maintaining lysosomal biogenesis and autophagy regulation. Enhancing the transcriptional function of TFEB upregulates the levels of lysosomal genes, lysosomal hydrolase genes and some autophagy-related genes. The decreased lysosomal function and disrupted autophagic flux suggested that the levels of the upstream factor, TFEB, might also be disrupted. Using a nuclear and cytoplasmic protein extraction kit, we detected the distribution of TFEB within the nucleus and cytoplasm of SGNs during the process of SGN degeneration. The results revealed that a disruption in TFEB distribution in the degenerated SGNs first appeared on the 5th day after ototoxic drug administration. TFEB levels gradually decreased in the nucleus and simultaneously increased in the cytoplasm (Figure 7(a,b)), which demonstrated that TFEB nuclear translocation was disrupted in degenerated SGNs.

### The MTOR inhibitor CCI-779 promoted TFEB shuttling into the nucleus in degenerated SGNs

The above results suggested that defective TFEB nuclear translocation was probably an important molecular component of the progression of SGN degeneration. Therefore, we considered that stimulating TFEB to translocate into the nucleus might increase the lysosomal levels in SGNs, thus, ameliorating the defective autophagy-lysosomal pathway and potentially preventing or alleviating SGN degeneration. The transcriptional regulation of the CLEAR genes requires TFEB to translocate into the nucleus; this activity is mainly determined by TFEB phosphorylation levels [26]. CCI-779, an analog of rapamycin, causes dephosphorylation of TFEB by inhibiting MTOR, which facilitates TFEB transport into the nucleus [27]. Therefore, after we determined that p-MTOR expression was significantly decreased following CCI-779 injection (Figure 7(e,f)), the distribution of TFEB in the cytoplasm and nucleus of degenerated SGNs was analyzed. The results revealed that compared with the negative control group, the experimental group presented with less cytoplasmic TFEB, while TFEB in the nucleus showed the opposite trend (Figure 7(c,d)). These results indicated that CCI-779 promoted TFEB shuttling from the cytoplasm into the nucleus of degenerated SGNs.

### Lysosomal biogenesis and autophagic flux in degenerated SGNs were restored after CCI-779 intervention

**Figure 8.**
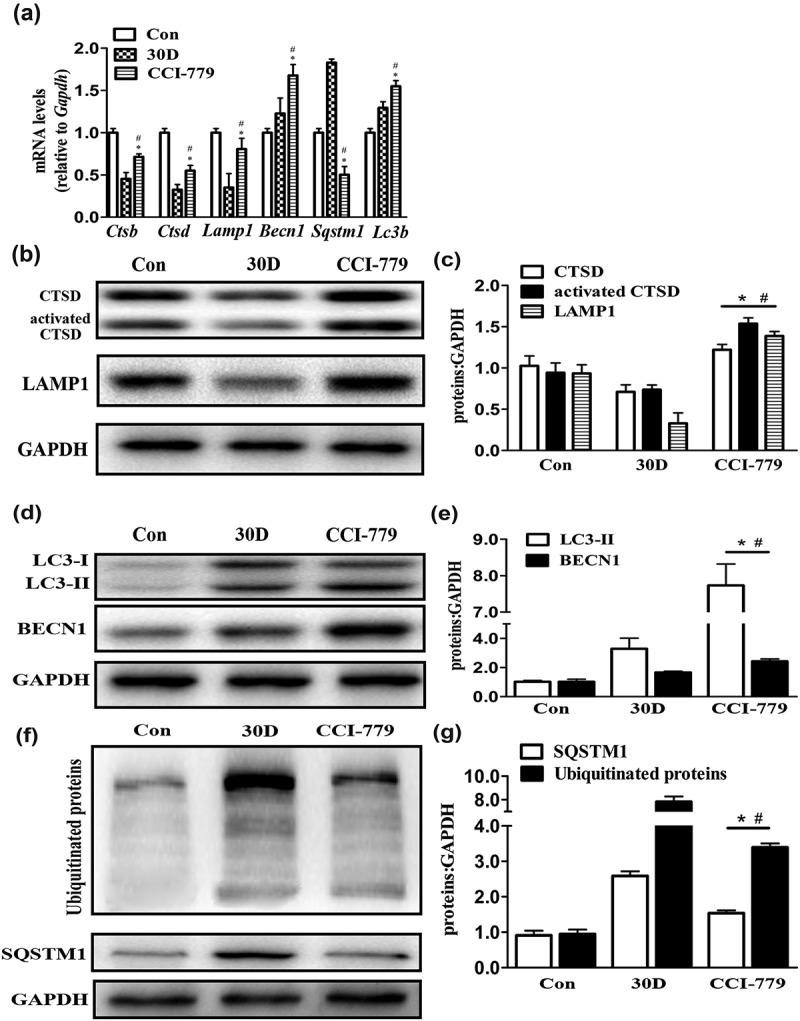
CCI-779 significantly rescued the impaired autophagy-lysosomal pathway in degenerated SGNs of mice. (a) The levels of *Ctsb, Ctsd*, and *Lamp1* and of the autophagic genes *Becn1* and *Lc3b* were significantly higher, and *Sqstm1* was lower in the experimental group than in the negative control group, which was determined by quantitative real-time PCR. (b and c) The LAMP1 and CTSD levels determined by western blotting were consistent with the quantitative real-time PCR results. (d and e) Compared with those in the negative control group, the LC3 and BECN1 levels in the experimental groups were significantly increased, as determined by western blot assays. (f and g) The western blot results revealed that the levels of the autophagic cargo receptor SQSTM1 and ubiquitinated proteins were decreased significantly in the experimental group compared with those in the negative control groups. *, the difference between the experimental group and the blank control group was significant (*P* < 0.05); #, the difference between the experimental group and the negative control group was significant (*P* < 0.05); CCI-779, experimental group; 30D, negative control group; Con, blank control group.

CCI-779 promotes TFEB nuclear translocation in degenerated SGNs, and TFEB has been reported to be a key molecule in regulating the autophagy-lysosomal pathway. Considering these results, we detected lysosomal biogenesis in degenerated SGNs to determine whether stimulating TFEB relocation to the nucleus increased lysosomal biogenesis and restored autophagic flux. The real-time PCR results revealed that the expression of key lysosomal genes *Lamp1, Ctsb* and *Ctsd* was significantly higher in the experimental group than the expression in the negative control group (Figure 8(a)). Moreover, western blotting analysis of LAMP1 and CTSD confirmed the above observations (Figure 8(b,c)). These results revealed that promoting TFEB translocation from the cytoplasm into the nucleus significantly enhanced lysosomal levels in degenerated SGNs. Next, we examined lipofuscin accumulation in degenerated SGNs using transmission electron microscopy and found that the lipofuscin area within the cytoplasm of degenerated SGNs was still greater in the cochleae of the experimental group than in the cochleae of the blank control group (wild-type mice injected with saline) but was significantly less than that in the cochleae of the negative control group (Fig. S1).

The detection of autophagy-related gene expression by real-time PCR showed that *Lc3b* expression was significantly higher in the experimental group than in the blank and negative control groups (*P* < 0.05). In addition, *Sqstm1* expression, which is a critical marker that reflects the degree of autophagic degradation, was significantly decreased in the experimental group compared to that in the blank control group (Figure 8(a)). The western blot assessment of SQSTM1 and ubiquitinated protein (Figure8(d-g)) levels was also consistent with the real-time PCR results. These results suggested that in degenerated SGNs the impaired autophagic flux was significantly rescued after CCI-779 was injected to promote TFEB nuclear translocation. These results indicated that after promoting TFEB nuclear translocation in degenerated SGNs in the cochleae of mice by administering CCI-779, lysosomal biogenesis and autophagic flux were partially restored.

### Amelioration of autophagy-lysosomal pathway disruption attenuated SGN and nerve fiber degeneration

**Figure 9.**
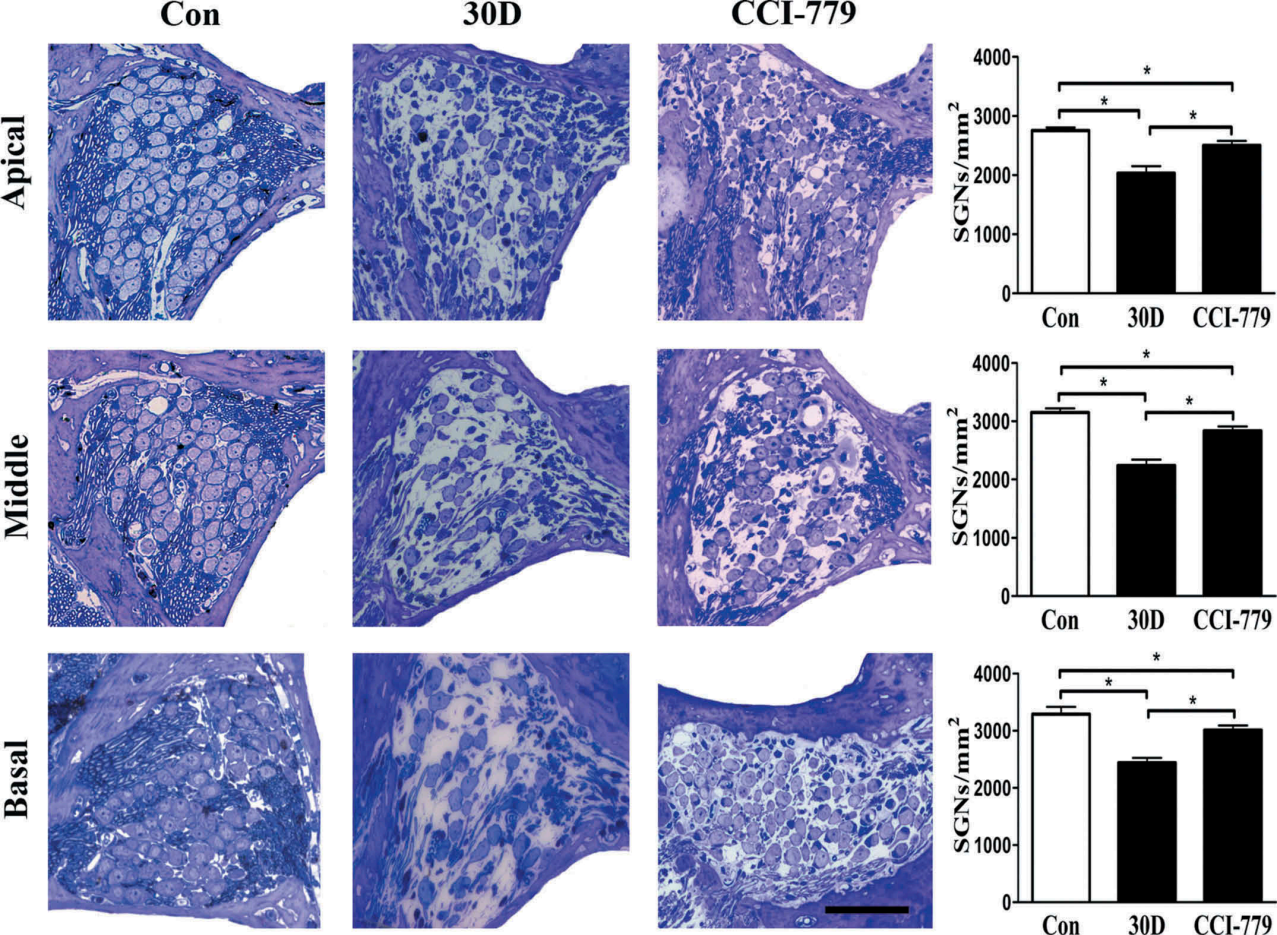
SGN degeneration was significantly attenuated with CCI-779 intervention. Light microscopy observation of toluidine blue staining revealed that SGN density in the apical, middle and basal turn of the experimental group was significantly higher than that of the negative control group but was still lower than that in the blank control group. *, the difference between the 2 groups was significant (*P* < 0.05); CCI-779, experimental group; 30D, negative control group; Con, blank control group. Scale bar: 50 µm.

**Figure 10.**
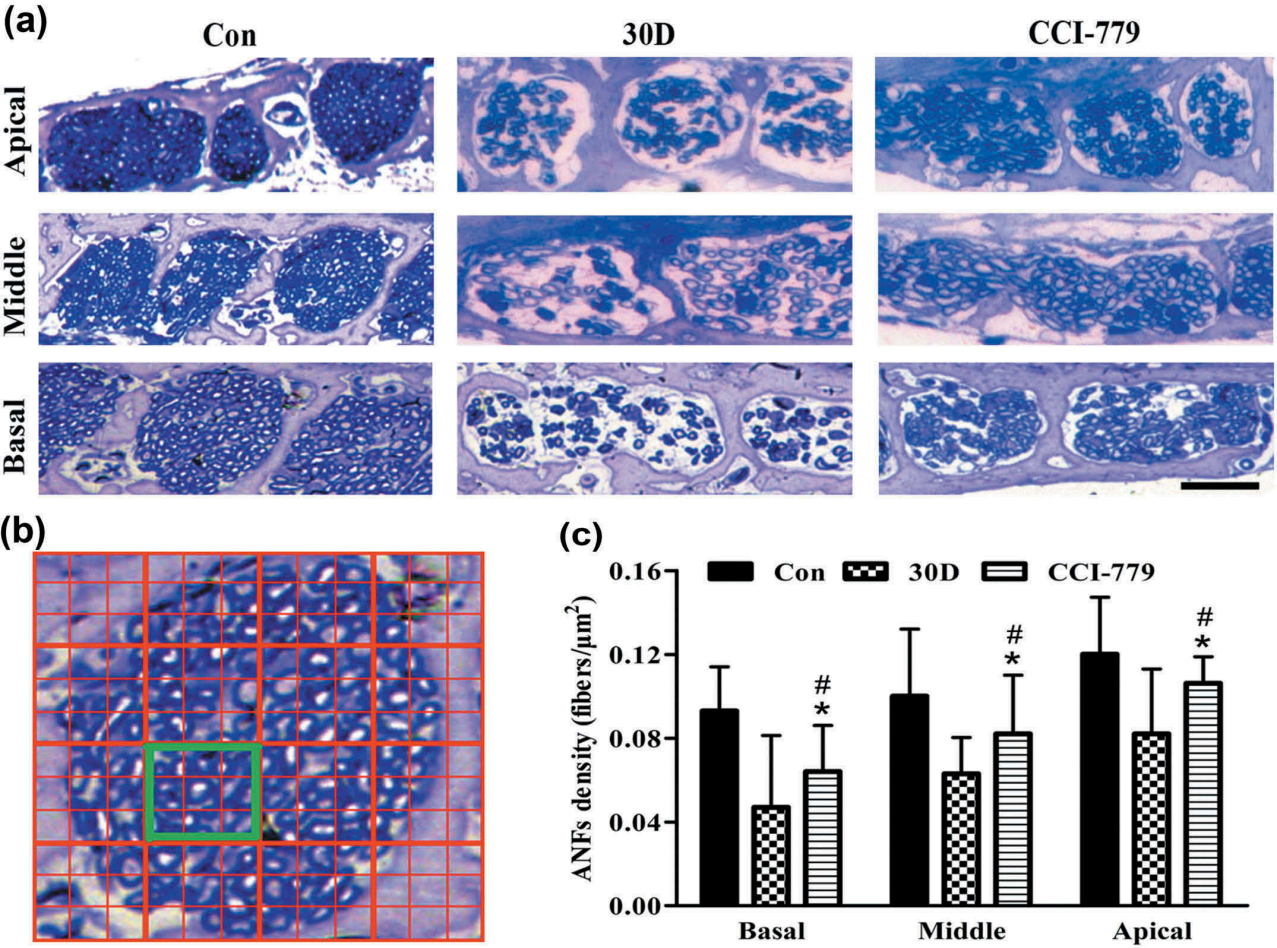
The degenerative rate of auditory nerve fibers was also significantly reduced with the CCI-779 intervention. (a and c) Compared with that of the negative control group, light microscopy observation of toluidine blue staining revealed that the nerve fiber density in the habenula perforata of the experimental group was significantly higher in the apical, middle and basal turns. (b) A diagram model of the calculated auditory nerve fiber density. The number of axons in the yellow box was counted. The size of the yellow box is 0.01 mm x 0.01 mm. The density of auditory nerve fibers (ANFs) was determined by the number of axons/100 μm^2^. *, the difference between the experimental group and the blank control group was significant (*P* < 0.05); #, the difference between the experimental group and the negative control group was significant (*P* < 0.05); CCI-779, experimental group; 30D, negative control group; Con, blank control group. Scale bar: 25 µm.

Because CCI-779 promoted TFEB nuclear translocation and rescued the autophagy-lysosomal pathway, we speculated that restoring autophagic function may alleviate SGN degeneration in mice. We simultaneously evaluated SGN and nerve fiber density in the habenula perforata with toluidine blue staining. The results revealed that the SGN density in the bottom, middle and apical turns of the cochlea of the experimental group was significantly greater (Figure 9) than that of the negative control group but was still lower than that of the blank control group. The same conclusion was also obtained from the detection of nerve fiber density (Figure 10). These results indicated that the MTOR inhibitor CCI-779 significantly attenuated SGN and nerve fiber degeneration in the SGN degeneration mouse model.

### Ameliorating autophagy-lysosomal pathway disruption reduced oxidative stress levels in degenerated SGNs

Oxidative stress is a crucial contributor to the development of neurodegenerative diseases [28–30] and is involved in sensorineural hearing loss induced by many factors such as noise [17], ototoxic drugs [31], radioactive radiation [32], ischemia [33], and aging [34]. Many antioxidants such as ebselen [35], N-acetylcysteine [17], and L-cysteine [36] have shown potential therapeutic effect on sensorineural hearing loss in several animal models. In 2017, *Lancet* reported a phase II randomized, double-blind trial using ebselen to treat patients with noise-induced hearing loss. The results from this trial demonstrated that ebselen prevents the temporary hearing threshold shift resulting from acute noise injury via enhancing GPX1 (glutathione peroxidase 1) activity [35]. Currently, antioxidative therapy for sensorineural hearing loss is mainly focused on reducing active oxygen release or increasing intracellular antioxidant capacity in the inner ear. However, conventional antioxidants are almost useless for eliminating oxidized proteins or severely damaged organelles [37]. The mechanism of the autophagy-lysosomal pathway tells us that autophagy can eliminate oxidized proteins, lipids, and DNA, as well as impaired mitochondria, thus preventing oxidative stress. The antioxidant capacity of autophagy is more effective and direct than compounds that inhibit specific oxidases or reactive oxygen species. Therefore, autophagy is an ideal means for antioxidant protection in neurodegenerative disease. Autophagy-lysosomal pathway impairment results in an accumulation of a large number of peroxidation proteins and lipids inside the cell soma, thereby triggering the death of neurons. Many experiments in vivo and in vitro have confirmed that the autophagy-lysosomal system is one of the basic antioxidant pathways. Therefore, we consider that autophagy upregulation may be helpful to reduce the level of oxidative stress, thus effectively protecting cells, especially in neurodegenerative diseases.

**Figure 11.**
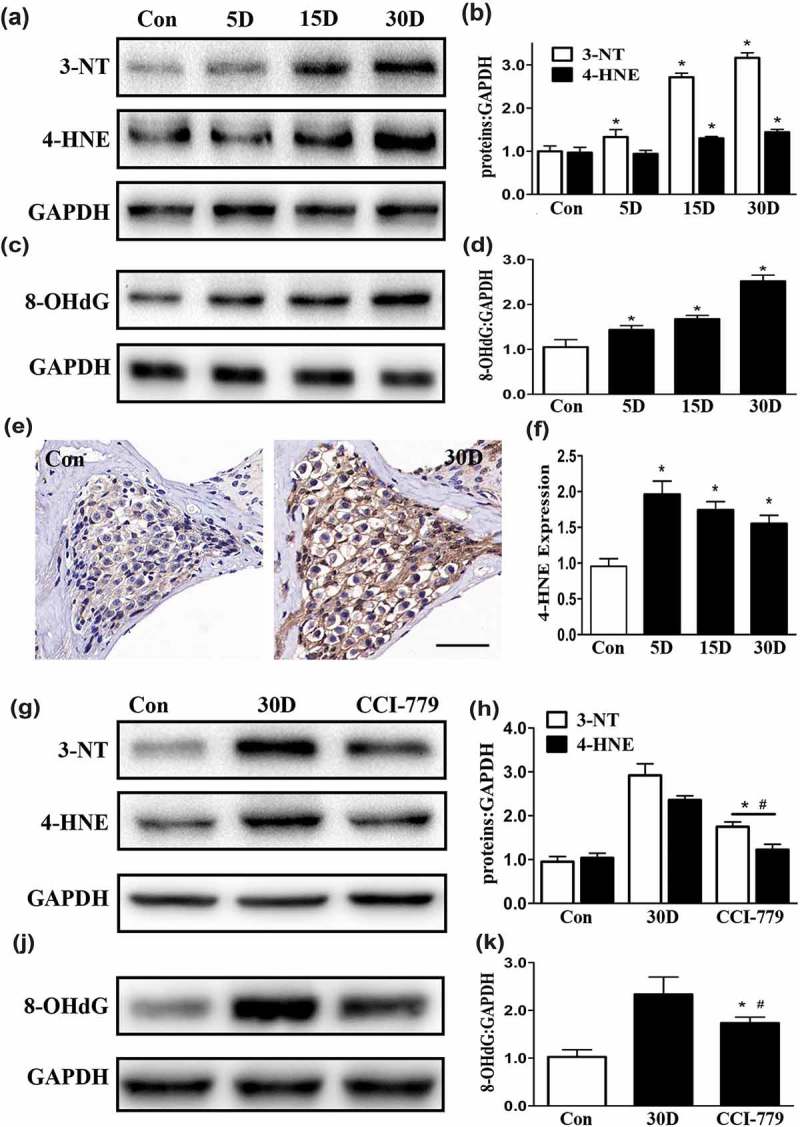
Oxidative stress in degenerated SGNs was diminished by the CCI-779 intervention. (a-d) The western blot results revealed that compared with those in the blank control group, the levels of 3-NT, 4-HNE, and 8-OHdG in the degenerated SGNs were significantly increased throughout the process of SGN degeneration. (e and f) The immunohistochemical 4-HNE staining results were also consistent with the western blot results. Images were taken from the middle turn of cochlea. (g-k) The 3-NT, 4-HNE and 8-OHdG levels in the SGNs of the experimental group were much lower than those in the negative control group but were still higher than those in the blank control group based on western blot. *, the difference between the experimental group and the blank control group was significant (*P* < 0.05); #, the difference between the experimental group and the negative control group was significant (*P* < 0.05); CCI-779, experimental group; 30D, negative control group; Con, blank control group. Scale bar: 50 µm.

When autophagy is defective, some iron-containing proteins in lipofuscins produce more ROS through Fenton chemistry to keep cells under persistent oxidative stress [38]. 3-Nitrotyrosine (3-NT), 4-hydroxynonenal (4–HNE), and 8-hydroxy-2ʹ-deoxyguanosine (8-OHdG) are useful markers to evaluate oxidative stress levels in the inner ear [17,22]. Therefore, we examined the changes in 3-NT, 4-HNE and 8-OHdG expression during the degenerative course of SGNs. The results showed that the levels of 3-NT, 4-HNE and 8-OHdG were significantly higher in degenerative SGNs than in blank controls, indicating a higher level of oxidative stress (Figure 11(a-d)). In addition, 4-HNE immunohistochemical staining demonstrated that the level of oxidative stress in the degenerated SGNs was consistent with the western blot observations (Figure 11(e,f), only show 4-HNE). These results indicated that the level of oxidative stress was remarkably increased in degenerated SGNs in the cochlea. Because the autophagy-lysosomal pathway may empower the ability to resist oxidative stress damage, we sought to determine whether oxidative stress was reduced in the degenerated SGNs after rescue of autophagy impairment. In other words, we examined whether the alleviation of SGN degeneration following the rescue of autophagy-lysosomal pathway disruption was associated with decreased oxidative stress. Thus, we detected the levels of 3-NT, 4-HNE and 8-OHdG after amelioration of autophagy impairment. The results revealed that the levels of 3-NT, 4-HNE and 8-OHdG in the experimental group were significantly lower than those in the negative control group (Figure11(g-k)). These results demonstrated that restoring the autophagy-lysosomal pathway reduced oxidative stress in degenerated SGNs.

## Discussion

Hearing loss is a common sensory neurological disorder in this noisy, engaged modern society. Approximately 5.3% of the world’s population (360 million people) suffers from hearing impairment, including 328 million adults and 32 million children [39]. SGNs in the cochlea are the first relay element in the hearing conduction pathway and deliver the electrical signals converted from sound stimulation by hair cells to the auditory center. There are approximately 32,000–41,000 SGNs in the human cochlea, 95% of which are type I SGNs that have myelin sheaths, and the remainder are type II SGNs [40]. The main pathological basis underlying sensorineural hearing loss is hair cell loss and retrograde SGN degeneration. For decades, researchers have focused on the regeneration of cochlear hair cells and have achieved remarkable success [41,42]. If hair cell regeneration is attainable, the survival of SGNs and their nerve endings appears to be particularly critical because the restoration of hearing function requires SGN nerve endings to innervate regenerated hair cells.

Cochlear implantation is an effective electronic biomimetic device that can replace damaged hearing cells by changing external sounds into electrical signals that can directly stimulate residual SGNs to send nerve impulses to the auditory center. Currently, due to their reliable effects, cochlear implants have allowed more than 300,000 hearing-impaired people to return to the ‘noisy’ world [43]. This success has allowed researchers to transfer their attention from trying to regenerate hearing cells to promoting SGN survival during the progression of sensorineural hearing loss. If the SGN degeneration process can be decelerated or alleviated, the effect of cochlear implants would be further improved, and a longer observation time would be provided to patients who are not appropriate candidates for cochlear implantation temporarily, especially elderly patients with age-related hearing loss.

Many previous studies have confirmed that a variety of common injurious factors that cause sensorineural hearing loss, such as cisplatin, aminoglycosides, diuretics and noise, mainly damage cochlear hair cells and not SGNs directly [1,44,45]. With the highest incidence, deafness caused by inherited genetic deficits accounts for approximately 60% of congenital sensorineural hearing loss. Among all known deafness genes, most of them are expressed in the scala media of the inner ear; only a few auditory neuropathy genes are in SGN terminals (http://hereditaryhearingloss.org/main.aspx?c=.HHH&n=87131). Therefore, most deafness genes primarily cause sensory epithelial cell damage, which is then followed by delayed degeneration of SGNs, finally resulting in hearing loss. Studies in recent years have indicated that hair cell survival may not be the only factor maintaining SGN survival, although SGN degeneration following hair cell loss is widely accepted. Zilberstein et al. [46], reported that adult mice harboring mutations in *Slc19a2* presented a phenotype of rapid inner hair cell death upon being fed a thiamine-deficient diet. Surprisingly, the afferent and efferent nerve terminals of the SGNs remained morphologically intact 3 months after inner hair cell loss. Recently, the Kurioka group reported a type of mouse specifically expressing diphtheria toxin receptors (DTRs) in its cochlear hair cells. This group found that when these specific mice were injected with diphtheria toxin, only hair cells were damaged, and no significant loss of SGNs could be observed for 2 months after drug administration [47]. These results and findings of Konstantina [4] suggested that SGN degeneration might require the damage of both hair cells and supporting cells. To simulate the pathological changes of most sensorineural hearing loss in the cochlea, our research group has been using kanamycin and furosemide to establish a mouse model of cochlear SGN and nerve-ending degeneration. Unlike gentamicin (another aminoglycoside drug), which damages hair cells and nerve fibers simultaneously [48], kanamycin does not directly damage mature SGN. With electron and light microscopy, in our previous studies, we found that kanamycin and furosemide destroyed the cochlear sensory epithelial cells of adult mice in a very short period and did not directly injure the soma of SGNs [19,20]. In our previous study about the re-innervation of the regenerated hair cells in the cochleae of chickens, the TEM results showed that the morphological structures of synapses beneath the injured hair cells were normal [44]. Ding et al. [49] recently demonstrated that the morphology and number of SGNs were also unchanged when kanamycin was administered to the cultured SGNs of adult mice, further confirming our observations. Therefore, we consider that the degenerative pattern of SGNs and their nerve endings in the cochlea of the mouse model established with kanamycin and furosemide is similar to the changes in SGNs and their nerve endings in the cochlea of the majority of sensorineural hearing loss cases.

Autophagy is an important cellular mechanism that maintains homeostasis in neurons and other non-mitotic cells. Many studies have discovered that autophagy dysfunction is involved in the occurrence and progression of neurodegenerative diseases [10–12,50]. Regarding the relationship between autophagy and the inner ear, previous studies have shown that autophagy plays a crucial role in the hair cell damage caused by ototoxic drugs, noise and other exogenous factors. When autophagy levels are increased in the inner ear, hair cell damage is reduced, which protects the auditory capacity of mice [18]. Compared with the autophagy levels in SAMR1 mice (senescence-accelerated mouse/resistance), those in SAMP8 mice (senescence-accelerated mouse/prone), which serve as a premature age-related hearing loss model [22], are significantly higher. Moreover, as SGNs develop and auditory function increases in young mice, the autophagy levels also increase in the mouse cochleae, indicating that autophagy may play a critical role in SGN development [15]. In pathomorphological terms, the degenerative pattern of SGNs and their nerve endings is similar to that of many neurodegenerative diseases [19,20,51,52]. However, to our knowledge, the connection between autophagy and SGN degeneration has not yet been elucidated. In the present study, we found that when SGN degeneration occurred following hair cell destruction, the lipofuscin area was substantially enlarged in neuron bodies. Since lipofuscin consists of debris from useless materials, such as peroxidated lipids and damaged proteins that are not fully digested by the autophagy-lysosomal system [21], the increased lipofuscin area in the degenerated SGNs indicates autophagy impairment [21,23]. This study is the first to report that autophagy impairment occurs during SGN degeneration.

According to previous reports on neurodegenerative diseases, autophagy dysfunction can be caused by various stages of autophagic flux, including decreased autophagic initiation [11,13,50,53], impaired autophagosome maturation [54], defective autophagosome fusion with lysosomes [55] and damaged lysosomal degradation [14,27]. Of course, autophagy arrest is not involved in every neurodegenerative disorder. In an ALS mouse model with mutated *Sod1*, neuronal damage is caused by autophagy-lysosomal pathway overactivation, based on the absence of lipofuscin [56]. According to our observations, degenerated SGNs have impaired autophagy degradation. The autophagic degradation capacity depends largely on the lysosomal capacity; thus, lysosomes are very important for neuronal survival. Normal lysosomal function requires sufficient hydrolases, normal lysosome activity and a suitable acidic environment. Lysosomal hydrolase impairment, lysosomal activity alterations [14,24,57,58], and pH variations [25,59] have been reported to contribute to neurodegenerative disease progression. In the present study, autophagy impairment in degenerated SGNs was correlated with limited levels of lysosomes and hydrolases. Further research is needed to determine whether lysosomal activity and/or the acidic environment are altered.

TFEB is a critical transcription factor that regulates lysosomal capacity and the autophagy pathway [26,60,61]. Recently, dysfunctional TFEB nuclear translocation was reported to induce deficient lysosomal capacity and autophagy arrest, which are associated with the development of HD, PD, AD and other neurodegenerative diseases [27,62,63]. Therefore, promoting TFEB translocation into the nucleus to restore autophagy-lysosomal pathway function may be a potential treatment strategy for neurodegenerative diseases [27,64,65]. Because of the impaired autophagy and significantly reduced lysosomal capacity in the autophagy-lysosomal pathway during SGN degeneration, we examined TFEB distribution in degenerated SGNs and found that TFEB was retained in the cytoplasm. After observing that TFEB was incapable of translocating to the nucleus, we ameliorated the disrupted autophagy-lysosomal pathway using pharmacological methods to translocate TFEB into the nucleus, and we found that the lysosome and hydrolase levels were partially recovered. Importantly, morphological examinations revealed that shuttling TFEB into the nucleus significantly relieved the extent of SGN and nerve fiber degeneration in the mouse cochleae. Because deteriorating SGNs are difficult to regenerate, our findings have promising theoretical and practical significance for the prevention and treatment of sensorineural hearing loss.

TFEB-mediated nuclear translocation dysfunction results in autophagy impairment in degenerated SGNs; therefore, determining how to effectively translocate TFEB into the nucleus is of great importance for delaying or preventing SGN degeneration in mice. Under normal conditions, phosphorylated TFEB is mainly located in the cytoplasm. When influenced by external stress factors, TFEB is dephosphorylated and shuttled into the nucleus to initiate the transcription of downstream genes that regulate the autophagy-lysosomal pathway. The important phosphorylation sites on TFEB are Ser142, Ser211 and the C-terminal sequences [26]. According to previous reports, there are four approaches for activating TFEB nuclear translocation: 1) PPARGC1A/PGC-1α [66] enhances the transcriptional activity of TFEB in the nucleus to upregulate target genes more effectively. 2) MAPK1/ERK2 (mitogen-activated protein kinase 1) [26] mediates the phosphorylation of Ser142 of TFEB to control its subcellular localization. 3) TNFRSF11A/RANK (tumor necrosis factor receptor superfamily, member 11a, NFKB activator) [67] promotes the translocation of TFEB into the nucleus and increases its transcriptional activity, as PRKCB/PKCβ phosphorylates the C-terminal region of TFEB. 4) Inhibitors of MTOR (mechanistic target of rapamycin kinase) can also be used [60,68,69]. In the present study, we used a pharmacological method to facilitate TFEB translocation into the nucleus by inhibiting MTOR. Regulating MTOR signaling to facilitate TFEB translocation into the nucleus is the most efficient method and is a common approach. More importantly, many studies have confirmed that rapamycin, a typical MTOR inhibitor, delays the progression of some neurodegenerative diseases [18,27,70].

MTOR is composed of MTORC1 and MTORC2. Normally, the complex is formed by V-ATPase and activated small RRAG GTPases, which recruit MTORC1 to the lysosomal membrane to activate MTORC1. Activated MTORC1 phosphorylates Ser142 of TFEB, causing TFEB to localize to the cytoplasm [60,69]. When an MTOR inhibitor is used to suppress MTORC1, the inactivated MTORC1 cannot phosphorylate TFEB; therefore, TFEB is shuttled into the nucleus from the cytoplasm. CCI-779, a rapamycin derivative that has already been approved for clinical practice by the Food and Drug Administration (FDA) and by the European Medicines Agency (EMA), can effectively activate TFEB nuclear translocation. In animal models of HD [70] and PD [27], some researchers have also used CCI-779 to promote TFEB translocation into the nucleus of damaged neurons. The results of the present study showed that CCI-779 was capable of significantly stimulating TFEB translocation into the nucleus in degenerated SGNs to ameliorate autophagy dysfunction, ultimately reducing the degree of SGN degeneration in mice.

Hearing loss induced by noise [71], ototoxic drugs [36], or age-related factors [22] is always associated with increased oxidative stress in the SGNs of the mouse cochleae, which eventually contributes to SGN apoptosis. With the help of antioxidative drugs, SGN damage and hearing impairment can be reduced, which suggests that reducing oxidative stress levels in SGNs is a beneficial strategy for protecting SGNs [72]. Due to its ability to eliminate superoxide and damaged organelles, the autophagy-lysosomal system empowers the antioxidative capacity [30]. In theory, if autophagy impairment is rescued, the degree of oxidative stress should decrease. To determine whether oxidative levels in degenerated SGNs decrease after CCI-779 treatment, we analyzed the levels of 3-NT, 4-HNE and 8-OHdG, which are widely recognized oxidative stress markers in the inner ear [17]. In our observations, 3-NT, 4-HNE and 8-OHdG levels were significantly decreased in degenerated SGNs after autophagic flux was restored, indicating that restoring autophagy function to alleviate SGN degeneration might be related to a reduction in oxidative stress.

## Conclusion

In the present study, we found that during the process of SGN degeneration after sensory epithelial cell loss, the lipofuscin area and oxidative stress were increased, TFEB was retained in the cytoplasm of SGNs, and the autophagy-lysosomal pathway was impaired. CCI-779 promoted TFEB translocation into the nucleus by inhibiting the MTOR pathway, ameliorating the disrupted autophagy-lysosomal pathway, decreasing oxidative stress and ultimately alleviating SGN degeneration in mice.

## Materials and methods

### Animals

The experimental animals, C57BL/6J mice (aged 8–10 weeks), were purchased from the experimental animal center of the Shanghai Institute of the Chinese Biological Sciences Academy of Sciences. None of the animals had a history of ototoxic damage or noise exposure, and all were housed under standard laboratory conditions. The animal’s procedures were approved by the Ethics Committee of Xinhua Hospital Affiliated Shanghai Jiao Tong University School of Medicine (Shanghai, China).

### Mouse model of SGN degeneration

The mice were randomly divided into 2 groups. The SGN degenerative group (experimental group) was subcutaneously injected with 1 g/kg kanamycin sulfate (Sigma-Aldrich, E004000), and after 30 ~ 45 min, 0.4 g/kg furosemide (Tianjin Pharmaceutical Group, H12020527; 10 mg/ml) was injected intraperitoneally [19,20,51]. Mice in the experimental group were subgrouped on the 5th, 15th, and 30th day after drug administration, with 16 mice in each subgroup. Sixteen mice that were the same age served as the blank control group and were simultaneously treated with equal doses of saline by subcutaneous and intraperitoneal injections.

### Preparation of cochlear tissue

The mice were sacrificed, and cochleae were quickly removed from the temporal bone. The cochleae were instantly transferred to a dish containing ice-cold phosphate-buffered saline (PBS; sangon biotech, E607008-0500). Then, the stapes bone was removed, and a small hole was drilled into the top of the cochlea. Next, 4% paraformaldehyde (Aladdin, C104190) was slowly infused into the cochleae overnight at 4°C. The next day, paraformaldehyde was replaced with 10% EDTA (Aladdin, E116428), and the cochleae were stored at room temperature for 1 week. After decalcification and dehydration, 6 cochlear samples from each subgroup were embedded in tissue freezing medium (sakura, 4583). The frozen sections (7 µm) were obtained using a Leica cryostat (Leica, CM1850).

### Cochlear HE staining

Two cochlear samples were obtained at each time point according to the above method. After the gradient ethanol dehydration procedure (75%, 85%, 90%, 95% and 100% ethanol), xylene was added, then the samples were immersed in wax for 2 h. Paraffin-embedded, 3-μm-thick sections were cut parallel to the modiolus. Sections that contained 4 organs of Corti were retained. The obtained sections were microwaved for 30 min at 50°C, then dewaxed by xylene and alcohol in a serial gradient and stained using a hematoxylin and eosin solution (Beyotime, C0105).

### Hair cell counting

At each time point, the middle turn of Corti was carefully obtained from the decalcified cochleae and was prepared for immunofluorescence staining as we previously described [19]. After the tissue was blocked in 0.1 M PBS containing 10% donkey serum (Jackson, 017–000-121) with 0.3% Triton X-100 (Sigma, 9002–93-1) at room temperature for 1 h, it was incubated with a rabbit anti-MYO7A antibody (1: 300; Proteus Bioscience, 25–6790) at 4°C overnight. After the tissue was washed with 0.1 M PBS, the donkey anti-rabbit Alexa Fluor 594 secondary antibody (Jackson, 711–585-152; 1: 500) was added to the tissue for 1 h at room temperature. Finally, we calculated the percentage of surviving hair cells with MYO7A-positive staining under a confocal microscope (Zeiss, LSM710).

### Toluidine blue staining and lipofuscin area calculation

Six cochlear samples from each subgroup were fixed in 2.5% glutaraldehyde (Sigma-Aldrich, G7651) overnight at 4°C, decalcified, dehydrated using a gradient elution of ethanol and acetone solutions, and finally embedded in epoxy resin parallel to the modiolus. Continuous sections were cut (2-µm thick) until the modiolus, which contained 4 organs of Corti and all turns of the SGNs, was visible. The chosen sections were stained with toluidine blue and selected for counting under a light microscope. After the SGNs of the Rosenthal canal were located, 50 ~ 60-nm ultrathin sections were sliced with an ultramicrotome (Leica, UC7), stained with 3% uranyl acetate and lead citrate, and observed with a transmission electron microscope (Japan Electronics Company Limited, JEM-1200EX) at different time points. In each sample, 30 spiral ganglion neurons with obvious nuclei (n ≥ 5) were randomly selected and outlined (red circle, Figure 2(a)), and the area of the cytoplasm and lipofuscin granules was calculated using ImageJ software (NIH, Bethesda, MD, USA). Each neuron with lipofuscin granules was expressed as a percentage of the area of the cytoplasm [19].

### Determining SGN and auditory nerve fiber density

From every 10 continuous, semi-thin sections, one section was selected. Then, 5 sections per subgroup were stained with toluidine blue and were observed under a light microscope (n ≥ 4). ImageJ software was used to manually outline the Rosenthal canal area, and the SGNs that had obvious nuclei were counted as we have done previously [19,51]. The SGN density was calculated according to the number of SGNs per area (mm^2^). At the same time, the target sections were preserved by following the appearance order of habenula perforata. The chosen section was separated by 20 μm, and each turn of every 3 cochlear sections was analyzed (n > 3).

### Immunofluorescence and immunohistochemistry staining

Frozen sections that had an intact modiolus were selected for immunofluorescence staining. The sections were blocked with 10% donkey serum (Jackson, 017–000-121) and 0.3% Triton X-100 (Sigma-Aldrich, 9002–93-1) in 0.1 M PBS and then incubated with rabbit anti-LC3 (Novus, NB910-404; 1:300) overnight at 4°C. After the sections were washed 3 times with 0.1 M PBS, they were incubated with the secondary antibody donkey anti-rabbit Alexa Fluor 594 (Jackson, 711–585-152; 1: 500) at 37°C in the dark for 30 min. Finally, the sections were stained with DAPI (Beyotime, C1002; 1:1000) for 5 min after being washed and mounted with anti-fade reagent (Invitrogen, S36940). For immunohistochemical staining, the paraffin sections were heated in a microwave at 67°C for 2 h and then deparaffinized in a xylene and ethanol gradient. After the sections were washed 3 times with 0.1 M PBS, they were incubated with 3.0% H_2_O_2_ for 30 min at room temperature. Next, the sections were incubated with the primary 4-HNE (Abcam, ab46545; 1:50) antibody overnight at 4°C. The secondary horseradish peroxidase-labeled goat anti-rabbit IgG antibody (Beyotime, A0208, 1:500) was added for 1 h, and finally, the sections were stained with 3,3N-diaminobenzidine (Beyotime, P0203). The SGNs were observed under a confocal microscope (Zeiss, LSM710). The laser power intensity was set ranging from 15% to 30%, and the PMT gain range was within 300. The fluorescent pictures were obtained under the same laser intensity and PMT gain. For the negative control, the primary antibodies were replaced with 0.1 M PBS.

### Western blot and quantitative real-time PCR

To ensure that the extracts were mainly from SGNs, we removed the basilar membrane and stria vascularis as well as other tissues and collected only the modioli of the cochlea from 6 mice in each subgroup in pre-chilled 0.1 M PBS using a stereomicroscope (ZEISS, Stemi-2000). The modioli were dissolved in 100 μl of RIPA lysis buffer (PMSF:RIPA = 1:100; Beyotime, ST506, P0013B), pulverized with an ultrasonic pulverizer and centrifuged at 14,200 × g for 5 min; finally, the supernatant was collected. SDS-PAGE sample loading buffer was added to the supernatant for denaturation, and the samples were stored at −80°C. The cytoplasmic and nuclear proteins were extracted using a Nuclear and Cytoplasmic Protein Extraction kit (Thermo Fisher Scientific, 78833) according to the manufacturer’s protocol. Western blot and quantitative real-time PCR were performed as previously described [19,51]. The primary antibodies for western blot targeted TFEB, LC3 (Novus, NBP1-30908, NB910-40435; 1:500), 3-nitrotyrosine (Millipore, 05–233; 1:1000), BECN1, SQSTM1, ubiquitin (Novus, NB500-249, NBP1-48320, MAB8595; 1:1000), LAMP1 (Sigma-Aldrich, SAB3500285; 1:200), CTSD/cathespin D (Cell Signaling Technology, 2284; 1:300), MTOR, p-MTOR (Cell Signaling Technology, 2972S, 2971; 1:1000), and GAPDH (Beyotime, AG019; 1:1000). The primers for quantitative real-time PCR are listed in Table 1.

**Table 1.** Primers used for RT-PCR.

	Forward Primer	Reverse Primer	
Target Genes	(5ʹ to 3ʹ)	(5ʹ to 3ʹ)	Source
Becn1	GGAGAGAAGAGGAGCCAGGT	TGTTGCCTCCACTGAACTTG	PrimerBank
Lc3b	TTCTTCCTCCTGGTGAATGG	GTGGGTGCCTACGTTCTCAT	PrimerBank
Sqstm1	TGAAACATGGACACTTTGGCT	ACATTGGGATCTTCTGGTGGA	PrimerBank
Lamp1	AACCCCAGTGTGTCCAAGTA	GCTGACAAAGATGTGCTCCT	PrimerBank
Ctsb	GAAGAAGTCGTGTGGCACTG	GTTCGGTCAGAAATGGCTTC	PrimerBank
Ctsd	AGGTGAAGGAGCTGCAGAAG	ATTCCCATGAAGCCACTCAG	PrimerBank
Gapdh	GGTGAAGGTCGGTGTGAACG	CTCGCTCCTGGAAGATGGTG	PrimerBank

### CCI-779 preparation and experimental animal organization

CCI-779 (MedChem Express, HY-50910) was dissolved in pure ethanol at a final concentration of 50 mg/ml and was stored at −20°C. The drugs were diluted in Tween 80 (Sigma-Aldrich, 9005–65-6; 5%), PEG 400 (Sigma-Aldrich, 25322–68-3; 5%) and 0.15 M NaCl to a final concentration of 20 mg/ml before injection.

Forty mice from the mouse model of SGN degeneration were randomly divided into 2 groups: the experimental group and the negative control group. There were 20 mice in each group. The experimental group of mice was intraperitoneally injected with 20 mg/kg of CCI-779 3 times a week for 4 weeks beginning 1 day after receiving the ototoxic drugs. For the mice in the negative control group, CCI-779 administration was replaced with an equal injection volume of saline at the same time point. The blank control group, consisting of 12 normal mice of the same age, was simultaneously injected with equal doses of saline only. All mice in the experimental and control groups were sacrificed 1 day after the final injection; in other words, all mice were sacrificed on the 30th day after the SGN degeneration model mice received the ototoxic drug injections.

### Statistical analysis

All statistical analysis results are expressed as the means ± standard deviation (SD), and the analysis was performed using SPSS software (v 19.0; SPSS Inc., Chicago, IL, USA). Comparisons between 2 different time points were made using Student’s t-tests. The differences in the western blot results, densities of SGN and auditory nerve fibers, lipofuscin areas and real-time PCR data were compared by one-way ANOVA, followed by Dunnett’s post hoc test. P values < 0.05 were considered statistically significant.

## References

[1] Staecker H, Gabaizadeh R, Federoff H, et al Brain-derived neurotrophic factor gene therapy prevents spiral ganglion degeneration after hair cell loss. Otolaryngol Head Neck Surg. 1998 7;119(1):7–13. PubMed PMID: 9674508; eng. 9674508 10.1016/S0194-5998(98)70194-9

[2] Nadol JB Jr., Young YS, Glynn RJ. Survival of spiral ganglion cells in profound sensorineural hearing loss: implications for cochlear implantation. Ann Otol Rhinol Laryngol. 1989 6;98(6):411–416. PubMed PMID: 2729822; eng. 2729822 10.1177/000348948909800602

[3] Reiss LA, Turner CW, Karsten SA, et al Consonant recognition as a function of the number of stimulation channels in the Hybrid short-electrode cochlear implant. J Acoust Soc Am. 2012 11;132(5):3406–3417. PubMed PMID: 23145621; PubMed Central PMCID: PMCPMC3505213. eng. 23145621 10.1121/1.4757735 PMC3505213

[4] Stankovic K, Rio C, Xia A, et al Survival of adult spiral ganglion neurons requires erbB receptor signaling in the inner ear. J Neurosci. 2004 10 6;24(40):8651–8661. PubMed PMID: 15470130; eng. 15470130 10.1523/JNEUROSCI.0733-04.2004 PMC6729966

[5] Miller JM, Chi DH, O’Keeffe LJ, et al Neurotrophins can enhance spiral ganglion cell survival after inner hair cell loss. Int J Dev Neurosci. 1997 7;15(4–5):631–643. PubMed PMID: 9263039; eng. 9263039 10.1016/s0736-5748(96)00117-7

[6] Fukui H, Wong HT, Beyer LA, et al BDNF gene therapy induces auditory nerve survival and fiber sprouting in deaf Pou4f3 mutant mice. Sci Rep. 2012;2:838 PubMed PMID: 23150788; PubMed Central PMCID: PMCPMC3495341. eng. 23150788 10.1038/srep00838 PMC3495341

[7] Shepherd RK, Coco A, Epp SB. Neurotrophins and electrical stimulation for protection and repair of spiral ganglion neurons following sensorineural hearing loss. Hear Res. 2008 8;242(1–2):100–109. PubMed PMID: 18243608; PubMed Central PMCID: PMCPMC2630855. eng. 18243608 10.1016/j.heares.2007.12.005 PMC2630855

[8] Leake PA, Hradek GT, Snyder RL Chronic electrical stimulation by a cochlear implant promotes survival of spiral ganglion neurons after neonatal deafness. J Comp Neurol. 1999 10 4;412(4):543–562. PubMed PMID: 10464355; eng. 10464355 10.1002/(sici)1096-9861(19991004)412:4<543::aid-cne1>3.0.co;2-3

[9] Corrales CE, Pan L, Li H, et al Engraftment and differentiation of embryonic stem cell-derived neural progenitor cells in the cochlear nerve trunk: growth of processes into the organ of Corti. J Neurobiol. 2006 11;66(13):1489–1500. PubMed PMID: 17013931; PubMed Central PMCID: PMCPMC2040047. eng. 17013931 10.1002/neu.20310 PMC2040047

[10] Nixon RA, Yang DS Autophagy and neuronal cell death in neurological disorders. Cold Spring Harb Perspect Biol. 2012 10 01;4(10):a008839–a008839. PubMed PMID: 22983160; PubMed Central PMCID: PMCPMC3475163. Eng. 22983160 10.1101/cshperspect.a008839 PMC3475163

[11] Pickford F, Masliah E, Britschgi M, et al The autophagy-related protein beclin 1 shows reduced expression in early Alzheimer disease and regulates amyloid beta accumulation in mice. J Clin Invest. 2008 6;118(6):2190–2199. PubMed PMID: 18497889; PubMed Central PMCID: PMCPMC2391284. Eng. 18497889 10.1172/JCI33585 PMC2391284

[12] Pan T, Kondo S, Le W, et al The role of autophagy-lysosome pathway in neurodegeneration associated with Parkinson’s disease. Brain. 2008 8;131(Pt 8):1969–1978. PubMed PMID: 18187492; Eng. 18187492 10.1093/brain/awm318

[13] Shibata M, Lu T, Furuya T, et al Regulation of intracellular accumulation of mutant Huntingtin by Beclin 1. J Biol Chem. 2006 5 19;281(20):14474–14485. PubMed PMID: 16522639; eng. 16522639 10.1074/jbc.M600364200

[14] Dehay B, Martinez-Vicente M, Caldwell GA, et al Lysosomal impairment in Parkinson’s disease. Mov Disord. 2013 6;28(6):725–732. PubMed PMID: 23580333; Eng. 23580333 10.1002/mds.25462 PMC5131721

[15] de Iriarte Rodriguez R, Pulido S, Rodriguez-de la Rosa L, et al Age-regulated function of autophagy in the mouse inner ear. Hear Res. 2015 12;330(Pt A):39–50. PubMed PMID: 26235979; eng. 26235979 10.1016/j.heares.2015.07.020

[16] Zuo WQ, Hu YJ, Yang Y, et al Sensitivity of spiral ganglion neurons to damage caused by mobile phone electromagnetic radiation will increase in lipopolysaccharide-induced inflammation in vitro model. J Neuroinflammation. 2015;12:105 PubMed PMID: 26022358; PubMed Central PMCID: PMCPMC4458026. eng. 26022358 10.1186/s12974-015-0300-1 PMC4458026

[17] Yuan H, Wang X, Hill K, et al Autophagy attenuates noise-induced hearing loss by reducing oxidative stress. Antioxid Redox Signal. 2015 5 20;22(15):1308–1324. PubMed PMID: 25694169; PubMed Central PMCID: PMCPMC4410759. eng. 25694169 10.1089/ars.2014.6004 PMC4410759

[18] Fang B, Xiao H Rapamycin alleviates cisplatin-induced ototoxicity in vivo. Biochem Biophys Res Commun. 2014 6 13;448(4):443–447. PubMed PMID: 24796670; eng. 24796670 10.1016/j.bbrc.2014.04.123

[19] Hu H, Ye B, Zhang L, et al Efr3a Insufficiency Attenuates the Degeneration of Spiral Ganglion Neurons after Hair Cell Loss. Front Mol Neurosci. 2017;10:86 PubMed PMID: 28424585; PubMed Central PMCID: PMCPMC5372784. eng. 28424585 10.3389/fnmol.2017.00086 PMC5372784

[20] Nie C, Hu H, Shen C, et al Expression of EFR3A in the mouse cochlea during degeneration of spiral ganglion following hair cell loss. PloS one. 2015;10(1):e0117345 PubMed PMID: 25622037; PubMed Central PMCID: PMCPMC4306511. Eng. 25622037 10.1371/journal.pone.0117345 PMC4306511

[21] Di Guardo G Lipofuscin, lipofuscin-like pigments and autofluorescence. Eur J Histochem. 2015;59(1):2485 PubMed PMID: 25820564; PubMed Central PMCID: PMCPMC4378218. eng. 25820564 10.4081/ejh.2015.2485 PMC4378218

[22] Menardo J, Tang Y, Ladrech S, et al Oxidative stress, inflammation, and autophagic stress as the key mechanisms of premature age-related hearing loss in SAMP8 mouse Cochlea. Antioxid Redox Signal. 2012 2 1;16(3):263–274. PubMed PMID: 21923553; eng. 21923553 10.1089/ars.2011.4037

[23] Terman A, Dalen H, Brunk UT Ceroid/lipofuscin-loaded human fibroblasts show decreased survival time and diminished autophagocytosis during amino acid starvation. Exp Gerontol. 1999 12;34(8):943–957. PubMed PMID: 10673148; Eng. 10673148 10.1016/s0531-5565(99)00070-4

[24] Moors T, Paciotti S, Chiasserini D, et al Lysosomal dysfunction and alpha-synuclein aggregation in Parkinson’s disease: diagnostic links. Mov Disord. 2016 6;31(6):791–801. PubMed PMID: 26923732; eng. 26923732 10.1002/mds.26562

[25] Ramirez A, Heimbach A, Grundemann J, et al Hereditary parkinsonism with dementia is caused by mutations in ATP13A2, encoding a lysosomal type 5 P-type ATPase. Nat Genet. 2006 10;38(10):1184–1191. PubMed PMID: 16964263; eng. 16964263 10.1038/ng1884

[26] Settembre C, Di Malta C, Polito VA, et al TFEB links autophagy to lysosomal biogenesis. Science (New York, NY). 2011 6 17;332(6036):1429–1433. PubMed PMID: 21617040; PubMed Central PMCID: PMCPMC3638014. eng. 10.1126/science.1204592 PMC3638014 21617040

[27] Decressac M, Mattsson B, Weikop P, et al TFEB-mediated autophagy rescues midbrain dopamine neurons from alpha-synuclein toxicity. Proc Natl Acad Sci USA. 2013 5 7;110(19):E1817–26. PubMed PMID: 23610405; PubMed Central PMCID: PMCPMC3651458. eng. 23610405 10.1073/pnas.1305623110 PMC3651458

[28] Pal R, Bajaj L, Sharma J, et al NADPH oxidase promotes Parkinsonian phenotypes by impairing autophagic flux in an mTORC1-independent fashion in a cellular model of Parkinson’s disease. Sci Rep. 2016 3 10;6:22866 PubMed PMID: 26960433; PubMed Central PMCID: PMCPMC4785399. Eng. 26960433 10.1038/srep22866 PMC4785399

[29] Coyle JT, Puttfarcken P Oxidative stress, glutamate, and neurodegenerative disorders. Science (New York, NY). 1993 10 29;262(5134):689–695. PubMed PMID: 7901908; eng. 10.1126/science.7901908 7901908

[30] Giordano S, Darley-Usmar V, Zhang J Autophagy as an essential cellular antioxidant pathway in neurodegenerative disease. Redox Biol. 2014;2:82–90. PubMed PMID: 24494187; PubMed Central PMCID: PMCPMC3909266. eng. 24494187 10.1016/j.redox.2013.12.013 PMC3909266

[31] He Z, Guo L, Shu Y, et al Autophagy protects auditory hair cells against neomycin-induced damage. Autophagy. 2017;13(11):1884–1904. PubMed PMID: 28968134; PubMed Central PMCID: PMCPMC5788479. eng. 28968134 10.1080/15548627.2017.1359449 PMC5788479

[32] Tan PX, Du SS, Ren C, et al Radiation-induced Cochlea hair cell death: mechanisms and protection. Asian Pac J Cancer Prev. 2013;14(10):5631–5635. PubMed PMID: 24289554; eng. 24289554 10.7314/apjcp.2013.14.10.5631

[33] Maetani T, Hakuba N, Taniguchi M, et al Free radical scavenger protects against inner hair cell loss after cochlear ischemia. Neuroreport. 2003 10 6;14(14):1881–1884. PubMed PMID: 14534440; eng. 14534440 10.1097/00001756-200310060-00025

[34] Fujimoto C, Yamasoba T Oxidative stresses and mitochondrial dysfunction in age-related hearing loss. Oxid Med Cell Longev. 2014;2014:582849 PubMed PMID: 25110550; PubMed Central PMCID: PMCPMC4106174. Eng. 25110550 10.1155/2014/582849 PMC4106174

[35] Kil J, Lobarinas E, Spankovich C, et al Safety and efficacy of ebselen for the prevention of noise-induced hearing loss: a randomised, double-blind, placebo-controlled, phase 2 trial. Lancet. 2017 9 2;390(10098):969–979. PubMed PMID: 28716314; eng. 28716314 10.1016/S0140-6736(17)31791-9

[36] Lu S, Fan Z, Xu W, et al L-cysteine attenuates peroxynitrite-elicited cytotoxicity to spiral ganglion neurons: possible relation to hearing loss. Neurol Res. 2011 11;33(9):935–941. PubMed PMID: 22080994; eng. 22080994 10.1179/1743132810Y.0000000027

[37] Sies H Oxidative stress: a concept in redox biology and medicine. Redox Biol. 2015;4:180–183. PubMed PMID: 25588755; PubMed Central PMCID: PMCPMC4309861. eng. 25588755 10.1016/j.redox.2015.01.002 PMC4309861

[38] Sohal RS, Brunk UT Lipofuscin as an indicator of oxidative stress and aging. Adv Exp Med Biol. 1989;266:17–26; discussion 27–9 PubMed PMID: 2486150; Eng. 10.1007/978-1-4899-5339-1_2 2486150

[39] Davis A, McMahon CM, Pichora-Fuller KM, et al Aging and Hearing Health: the Life-course Approach. Gerontologist. 2016 4;56(Suppl 2):S256–67. PubMed PMID: 26994265; eng. 26994265 10.1093/geront/gnw033 PMC6283365

[40] Nayagam BA, Muniak MA, Ryugo DK The spiral ganglion: connecting the peripheral and central auditory systems. Hear Res. 2011 8;278(1–2):2–20. PubMed PMID: 21530629; PubMed Central PMCID: PMCPMC3152679. eng. 21530629 10.1016/j.heares.2011.04.003 PMC3152679

[41] Izumikawa M, Minoda R, Kawamoto K, et al Auditory hair cell replacement and hearing improvement by Atoh1 gene therapy in deaf mammals. Nat Med. 2005 3;11(3):271–276. PubMed PMID: 15711559; eng. 15711559 10.1038/nm1193

[42] Mizutari K, Fujioka M, Hosoya M, et al Notch inhibition induces cochlear hair cell regeneration and recovery of hearing after acoustic trauma. Neuron. 2013 1 9;77(1):58–69. PubMed PMID: 23312516; PubMed Central PMCID: PMCPMC3573859. eng. 23312516 10.1016/j.neuron.2012.10.032 PMC3573859

[43] Zeng FG, Rebscher SJ, Fu QJ, et al Development and evaluation of the Nurotron 26-electrode cochlear implant system. Hear Res. 2015 4;322:188–199. PubMed PMID: 25281795; eng. 25281795 10.1016/j.heares.2014.09.013

[44] Xiang ML, Mu MY, Pao X, et al The reinnervation of regenerated hair cells in the basilar papilla of chicks after kanamycin ototoxicity. Acta Otolaryngol. 2000 10;120(8):912–921. PubMed PMID: 11200585; eng. 11200585 10.1080/00016480050218636

[45] Zimmermann CE, Burgess BJ, Nadol JB Jr. Patterns of degeneration in the human cochlear nerve. Hear Res. 1995 10;90(1–2):192–201. PubMed PMID: 8974997; eng. 8974997 10.1016/0378-5955(95)00165-1

[46] Zilberstein Y, Liberman MC, Corfas G Inner hair cells are not required for survival of spiral ganglion neurons in the adult cochlea. J Neurosci. 2012 1 11;32(2):405–410. PubMed PMID: 22238076; PubMed Central PMCID: PMCPMC3678770. eng. 22238076 10.1523/JNEUROSCI.4678-11.2012 PMC3678770

[47] Kurioka T, Lee MY, Heeringa AN, et al Selective hair cell ablation and noise exposure lead to different patterns of changes in the cochlea and the cochlear nucleus. Neuroscience. 2016 9 22;332:242–257. PubMed PMID: 27403879; PubMed Central PMCID: PMCPMC4969227. eng. 27403879 10.1016/j.neuroscience.2016.07.001 PMC4969227

[48] Ruan Q, Ao H, He J, et al Topographic and quantitative evaluation of gentamicin-induced damage to peripheral innervation of mouse cochleae. Neurotoxicology. 2014 1;40:86–96. PubMed PMID: 24308912; eng. 24308912 10.1016/j.neuro.2013.11.002

[49] Gao K, Ding D, Sun H, et al Kanamycin damages early postnatal, but not adult spiral ganglion neurons. Neurotox Res. 2017 11;32(4):603–613. PubMed PMID: 28656549; PubMed Central PMCID: PMCPMC5711550. eng. 28656549 10.1007/s12640-017-9773-2 PMC5711550

[50] Komatsu M, Waguri S, Chiba T, et al Loss of autophagy in the central nervous system causes neurodegeneration in mice. Nature. 2006 6 15;441(7095):880–884. PubMed PMID: 16625205; Eng. 16625205 10.1038/nature04723

[51] Hu H, Ma Y, Ye B, et al The role of Efr3a in age-related hearing loss. Neuroscience. 2016 11 17;341:1–8. PubMed PMID: 27867060; eng. 27867060 10.1016/j.neuroscience.2016.11.013

[52] Ruel J, Wang J, Rebillard G, et al Physiology, pharmacology and plasticity at the inner hair cell synaptic complex. Hear Res. 2007 5;227(1–2):19–27. PubMed PMID: 17079104; eng. 17079104 10.1016/j.heares.2006.08.017

[53] Hara T, Nakamura K, Matsui M, et al Suppression of basal autophagy in neural cells causes neurodegenerative disease in mice. Nature. 2006 6 15;441(7095):885–889. PubMed PMID: 16625204; eng. 16625204 10.1038/nature04724

[54] Nixon RA, Wegiel J, Kumar A, et al Extensive involvement of autophagy in Alzheimer disease: an immuno-electron microscopy study. J Neuropathol Exp Neurol. 2005 2;64(2):113–122. PubMed PMID: 15751225; eng. 15751225 10.1093/jnen/64.2.113

[55] Moreau K, Fleming A, Imarisio S, et al PICALM modulates autophagy activity and tau accumulation. Nat Commun. 2014 9 22;5:4998 PubMed PMID: 25241929; PubMed Central PMCID: PMCPMC4199285. eng. 25241929 10.1038/ncomms5998 PMC4199285

[56] Bandyopadhyay U, Nagy M, Fenton WA, et al Absence of lipofuscin in motor neurons of SOD1-linked ALS mice. Proc Natl Acad Sci USA. 2014 7 29;111(30):11055–11060. PubMed PMID: 25024188; PubMed Central PMCID: PMCPMC4121794. Eng. 25024188 10.1073/pnas.1409314111 PMC4121794

[57] Lwin A, Orvisky E, Goker-Alpan O, et al Glucocerebrosidase mutations in subjects with parkinsonism. Mol Genet Metab. 2004 1;81(1):70–73. PubMed PMID: 14728994; eng. 14728994 10.1016/j.ymgme.2003.11.004

[58] Qiao L, Hamamichi S, Caldwell KA, et al Lysosomal enzyme cathepsin D protects against alpha-synuclein aggregation and toxicity. Mol Brain. 2008 11 21;1:17 PubMed PMID: 19021916; PubMed Central PMCID: PMCPMC2600785. eng. 19021916 10.1186/1756-6606-1-17 PMC2600785

[59] Lee JH, Yu WH, Kumar A, et al Lysosomal proteolysis and autophagy require presenilin 1 and are disrupted by Alzheimer-related PS1 mutations. Cell. 2010 6 25;141(7):1146–1158. PubMed PMID: 20541250; PubMed Central PMCID: PMCPMC3647462. eng. 20541250 10.1016/j.cell.2010.05.008 PMC3647462

[60] Settembre C, Zoncu R, Medina DL, et al A lysosome-to-nucleus signalling mechanism senses and regulates the lysosome via mTOR and TFEB. Embo J. 2012 3 7;31(5):1095–1108. PubMed PMID: 22343943; PubMed Central PMCID: PMCPMC3298007. eng. 22343943 10.1038/emboj.2012.32 PMC3298007

[61] Lapierre LR, De Magalhaes Filho CD, McQuary PR, et al The TFEB orthologue HLH-30 regulates autophagy and modulates longevity in Caenorhabditis elegans. Nat Commun. 2013;4:2267 PubMed PMID: 23925298; PubMed Central PMCID: PMCPMC3866206. eng. 23925298 10.1038/ncomms3267 PMC3866206

[62] La Spada AR PPARGC1A/PGC-1alpha, TFEB and enhanced proteostasis in Huntington disease: defining regulatory linkages between energy production and protein-organelle quality control. Autophagy. 2012 12;8(12):1845–1847. PubMed PMID: 22932698; PubMed Central PMCID: PMCPMC3541300. eng. 22932698 10.4161/auto.21862 PMC3541300

[63] Polito VA, Li H, Martini-Stoica H, et al Selective clearance of aberrant tau proteins and rescue of neurotoxicity by transcription factor EB. EMBO Mol Med. 2014 7 28;6(9):1142–1160. PubMed PMID: 25069841; PubMed Central PMCID: PMCPMC4197862. eng. 25069841 10.15252/emmm.201303671 PMC4197862

[64] Xiao Q, Yan P, Ma X, et al Neuronal-Targeted TFEB accelerates lysosomal degradation of APP, reducing abeta generation and amyloid plaque pathogenesis. J Neurosci. 2015 9 02;35(35):12137–12151. PubMed PMID: 26338325; PubMed Central PMCID: PMCPMC4556784. eng. 26338325 10.1523/JNEUROSCI.0705-15.2015 PMC4556784

[65] Wang H, Wang R, Carrera I, et al TFEB overexpression in the P301S model of tauopathy mitigates increased PHF1 levels and lipofuscin puncta and rescues memory deficits. eNeuro. 2016 Mar-Apr;3(2). PubMed PMID: 27257626; PubMed Central PMCID: PMCPMC4876487. eng DOI:10.1523/eneuro.0042-16.2016 PMC4876487 27257626

[66] Tsunemi T, Ashe TD, Morrison BE, et al PGC-1alpha rescues Huntington’s disease proteotoxicity by preventing oxidative stress and promoting TFEB function. Sci Transl Med. 2012 7 11;4(142):142ra97 PubMed PMID: 22786682; PubMed Central PMCID: PMCPMC4096245. Eng. 10.1126/scitranslmed.3003799 PMC4096245 22786682

[67] Ferron M, Settembre C, Shimazu J, et al A RANKL-PKCbeta-TFEB signaling cascade is necessary for lysosomal biogenesis in osteoclasts. Genes Dev. 2013 4 15;27(8):955–969. PubMed PMID: 23599343; PubMed Central PMCID: PMCPMC3650231. eng. 23599343 10.1101/gad.213827.113 PMC3650231

[68] Martina JA, Chen Y, Gucek M, et al MTORC1 functions as a transcriptional regulator of autophagy by preventing nuclear transport of TFEB. Autophagy. 2012 6;8(6):903–914. PubMed PMID: 22576015; PubMed Central PMCID: PMCPMC3427256. eng. 22576015 10.4161/auto.19653 PMC3427256

[69] Pena-Llopis S, Vega-Rubin-de-Celis S, Schwartz JC, et al Regulation of TFEB and V-ATPases by mTORC1. Embo J. 2011 8 17;30(16):3242–3258. PubMed PMID: 21804531; PubMed Central PMCID: PMCPMC3160667. eng. 21804531 10.1038/emboj.2011.257 PMC3160667

[70] Ravikumar B, Vacher C, Berger Z, et al Inhibition of mTOR induces autophagy and reduces toxicity of polyglutamine expansions in fly and mouse models of Huntington disease. Nat Genet. 2004 6;36(6):585–595. PubMed PMID: 15146184; eng. 15146184 10.1038/ng1362

[71] Fetoni AR, De Bartolo P, Eramo SL, et al Noise-induced hearing loss (NIHL) as a target of oxidative stress-mediated damage: cochlear and cortical responses after an increase in antioxidant defense. J Neurosci. 2013 2 27;33(9):4011–4023. PubMed PMID: 23447610; Eng. 23447610 10.1523/JNEUROSCI.2282-12.2013 PMC6619303

[72] Mardones P, Hetz C Peroxisomes get loud: A redox antidote to hearing loss. Cell. 2015 11 5;163(4):790–791. PubMed PMID: 26544930; Eng. 26544930 10.1016/j.cell.2015.10.060

