# Supplementary material for: Restoring autophagic flux attenuates cochlear spiral ganglion neuron degeneration by promoting TFEB nuclear translocation via inhibiting MTOR
Source: Autophagy. 2019 Feb 1;15(6):998–1016. doi: 10.1080/15548627.2019.1569926 (PMC6526833; doi:10.1080/15548627.2019.1569926)
Supplement: Supplemental Material [file kaup-15-06-1569926-s001.zip › 1569926_supplementary information/supp. Figure Legend.docx]

**Supplements:**

**Figure S1.** The changes in SGN morphology and lipofuscins were attenuated after CCI-779 intervention. TEM observations revealed that in the experimental group, the SGNs were in better condition than those in the negative control group and were tightly surrounded by myelin sheaths, without obvious discontinuities. In contrast, SGNs were enveloped within a thin, loose myelin sheath after the 30th day of drug administration. In addition, the lipofuscin area in the SGNs of the experimental group was much lower than that in the negative control group but was still higher than that in the blank control group. *, the difference between the experimental group and the blank control group was significant (*P*<0.05); #, the difference between the experimental group and the negative control group was significant (*P*<0.05); CCI-779, experimental group; 30D, negative control group; Con, blank control group. Images of TEM were taken from the middle turn of cochlea. Scale bar: 1 µm.
