# Supplementary figures and images for: Restoring autophagic flux attenuates cochlear spiral ganglion neuron degeneration by promoting TFEB nuclear translocation via inhibiting MTOR
Source: Autophagy. 2019 Feb 1;15(6):998–1016. doi: 10.1080/15548627.2019.1569926 (PMC6526833; doi:10.1080/15548627.2019.1569926)

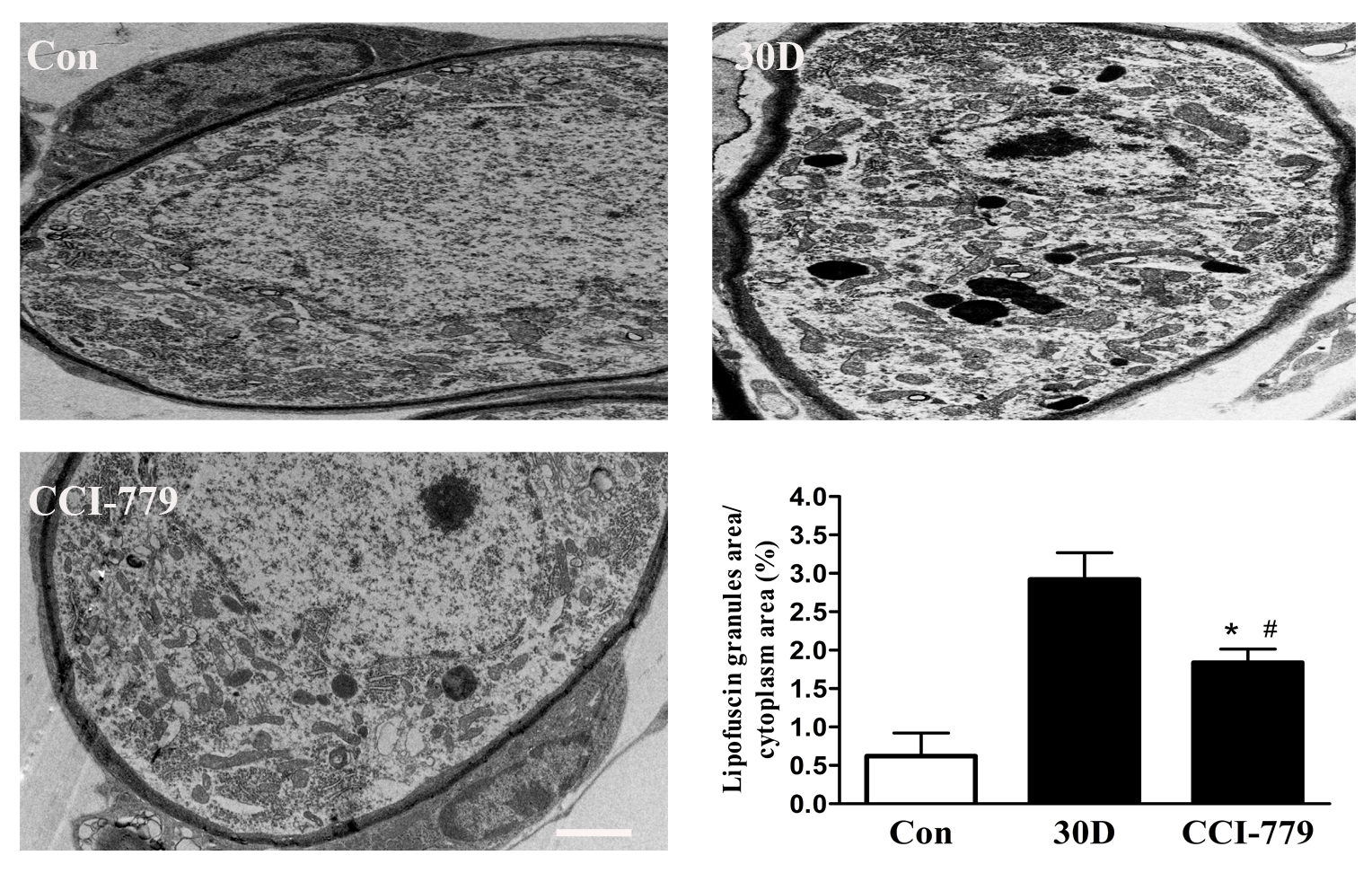

Supplement: Supplemental Material [file kaup-15-06-1569926-s001.zip › 1569926_supplementary information/FigS1R4.tif]
